# Evidence for the additivity of rare and common variant burden throughout the spectrum of intellectual disability

**DOI:** 10.1038/s41431-024-01581-3

**Published:** 2024-03-11

**Authors:** Lea Urpa, Mitja I. Kurki, Elisa Rahikkala, Eija Hämäläinen, Veikko Salomaa, Jaana Suvisaari, Riikka Keski-Filppula, Merja Rauhala, Satu Korpi-Heikkilä, Jonna Komulainen-Ebrahim, Heli Helander, Päivi Vieira, Johanna Uusimaa, Jukka S. Moilanen, Jarmo Körkkö, Tarjinder Singh, Outi Kuismin, Olli Pietiläinen, Aarno Palotie, Mark J. Daly

**Affiliations:** 1 Institute for Molecular Medicine Finland (FIMM), University of Helsinki, Helsinki, Finland; 2 The Stanley Center for Psychiatric Research, Broad Institute of MIT and Harvard, Cambridge, MA USA; 3 Psychiatric and Neurodevelopmental Genetics Unit, Massachusetts General Hospital, Boston, MA USA; 4 Research Unit of Clinical Medicine, University of Oulu, Oulu, Finland; 5 Medical Research Center, Oulu University Hospital, University of Oulu, Oulu, Finland; 6 Department of Clinical Genetics, Oulu University Hospital, Oulu, Finland; 7 Finnish Institute for Health and Welfare, Helsinki, Finland; 8 Oulu University Hospital, Center for Intellectual Disability Care, Oulu, Finland; 9 Intellectual Disability Department, Wellbeing services, County of Kainuu, Kajaani, Finland; 10 The Social Insurance Institution of Finland (KELA), Oulu, Finland; 11 Department of Pediatrics and Adolescent Medicine, Unit of Child Neurology, Oulu University Hospital, Oulu, Finland; 12 Department of Psychiatry, Columbia University, New York, NY 10032 USA; 13 New York Genome Center, New York, NY 10013 USA; 14 Mortimer B. Zuckerman Mind Brain and Behavioral Institute, Columbia University, New York, NY 10027 USA; 15 HiLIFE Neuroscience Center, University of Helsinki, Helsinki, Finland; 16 Analytic and Translational Genetics Unit, Department of Medicine, Massachusetts General Hospital, Boston, MA USA; 17 Department of Neurology, Massachusetts General Hospital, Boston, MA USA

**Keywords:** Neurodevelopmental disorders, Rare variants, Genetic variation

## Abstract

Intellectual disability (ID) is a common disorder, yet there is a wide spectrum of impairment from mild to profoundly affected individuals. Mild ID is seen as the low extreme of the general distribution of intelligence, while severe ID is often seen as a monogenic disorder caused by rare, pathogenic, highly penetrant variants. To investigate the genetic factors influencing mild and severe ID, we evaluated rare and common variation in the Northern Finland Intellectual Disability cohort (*n* = 1096 ID patients), a cohort with a high percentage of mild ID (*n* = 550) and from a population bottleneck enriched in rare, damaging variation. Despite this enrichment, we found only a small percentage of ID was due to recessive Finnish-enriched variants (0.5%). A larger proportion was linked to dominant variation, with a significant burden of rare, damaging variation in both mild and severe ID. This rare variant burden was enriched in more severe ID (*p* = 2.4e-4), patients without a relative with ID (*p* = 4.76e-4), and in those with features associated with monogenic disorders. We also found a significant burden of common variants associated with decreased cognitive function, with no difference between mild and more severe ID. When we included common and rare variants in a joint model, the rare and common variants had additive effects in both mild and severe ID. A multimodel inference approach also found that common and rare variants together best explained ID status (ΔAIC = 16.8, ΔBIC = 10.2). Overall, we report evidence for the additivity of rare and common variant burden throughout the spectrum of intellectual disability.

## Introduction

Intellectual disability (ID) is a common neurodevelopmental disorder, affecting approximately one percent of the global population [1]. Yet there exists a wide spectrum of impairment and comorbidity, ranging from profound disability requiring continuous support to mildly affected individuals who live independently in society. The study of ID genetics, mainly focused on more severe intellectual disabilities, has focused on the identification of causal variants in single genes or chromosomal abnormalities through identification by high-throughput sequencing technologies or array comparative genomic hybridization. This work has characterized severe intellectual disability as a heterogenous disorder where approximately 10% of genes in the genome could be disrupted to converge on profound impact in cognitive functioning and development [2].

In contrast, mild intellectual disability has been characterized as the low extreme of the general population distribution of intelligence, with evidence that factors influencing mild ID are similar to those influencing normal cognitive functioning and that mild ID is qualitatively distinct from severe intellectual disability [3, 4]. One assumption is that common genetic variants contribute to the more common diagnosis of mild intellectual disability, while more rare and penetrant variants are the cause of severe and profound ID. Yet recent studies have found that common genetic variation affects both the overall risk and clinical presentation in severe neurodevelopmental disorders previously considered to be monogenic [5, 6], and rare, damaging variants have been reported to contribute to reduced cognitive functioning among normal IQ individuals [7, 8].

To investigate the genetic factors influencing mild ID and disentangle them from those involved in severe intellectual disability, we recruited individuals with intellectual disability irrespective of their level of impairment in the Northern Finland Intellectual Disability Study (NFID; *n* = 1096 ID patients), resulting in a cohort enriched for mild intellectual disability (*n* = 550). We chose to recruit from Northern Finland because the small initial population of Finland and subsequent isolation have resulted in a founder effect, causing a relative increase in the frequency of certain variants (including damaging variants [9]). Later internal migration to the north of Finland resulted in a further population bottleneck, demonstrated by the increased concentration of Finnish Disease Heritage cases in the late settlement region [10].

We found that despite the enrichment of rare, damaging variants in Finland, recessive ID due to Finnish-enriched variants explains the etiology of only a small percentage of ID patients in the cohort. A larger proportion was explained by dominant variants, with a significant burden of rare, damaging variants in both mild and severe ID patients that was enriched in more severe ID, patients without a relative with ID, and in those with comorbid features associated with monogenic disorders. We found that the burden of common variants, while significant in both mild and more severe ID compared to controls, was indistinguishable between milder and more severe forms of affectedness. When we included both common and rare variants in the same model, we found that the rare and common variant burdens were additive in both mild and more severe ID. In all, we report evidence for the additivity of rare, damaging variant burden and common variant burden throughout the spectrum of mild to more severe intellectual disability.

## Methods

### NFID cohort and population controls

Individuals with intellectual disability (ICD-10 codes F70-79) or pervasive and specific developmental disorders (ICD-10 codes F80-89) of unknown etiology (*n* = 1096) were recruited from the three northernmost hospital districts in Finland for the Northern Finland Intellectual Disability (NFID) study from 2013 to 2019. DNA samples were obtained from peripheral blood or saliva, and all subjects were clinically examined by multi-professional teams. Detailed information on patient recruitment and examination can be found in the Supplementary Methods. All subjects and/or their legal guardians provided written informed consent to participate in the study, and the ethical committees of the Northern Ostrobothnia Hospital District and the Hospital District of Helsinki and Uusimaa approved the study. Exome sequencing and DNA array genotype data for population control individuals were obtained from the population-based health examination surveys FINRISK [11] and Health2000–2011 [12] via application to THL Biobank. Individuals in the FINRISK and Health2000–2011 studies that had known learning or psychiatric disorders as given in the study variables were excluded as controls.

### Exome sequencing and quality control

Samples from the NFID cohort, FINRISK, and Health2000–2011 were exome sequenced at the Broad Institute using either the Illumina (San Diego, California, USA) Nextera Rapid Capture Exome capture kit, the Agilent (Santa Clara, California, USA) SureSelect Human All Exon capture kit, or the Twist Bioscience (South San Francisco, California, USA) Human Core Exome capture kit, and sequenced on either Illumina HiSeq2000, 2500, 4000, X10, or NovaSeq 6000. NFID, FINRISK, and Health2000–2011 samples were jointly called with a collection of Finnish individuals as part of the Sequencing Initiative Suomi (SISU) study (www.sisuproject.fi). The sequence data processing and variant calling for this dataset has been previously described [13].

Exome sequencing variant, sample, and genotype quality control are described in detail in the supplementary methods. Kinship was calculated between all samples using King [14] and related individuals and population outliers were removed from analyses. All exome sequencing data quality control was performed using Hail [15] and executed in a Google Cloud Dataproc cluster. The exome sequencing data quality control pipeline is available at in a public repository at https://github.com/lea-urpa/exome_qc_library.

### Variant annotation

We annotated variants using the VEP [16] function (v.95) in Hail with the LOFTEE [17] VEP plugin to identify high-confidence loss-of-function likely pathogenic or pathogenic variants. We considered variant annotation only on the canonical ENSEMBL transcript, or the transcript with the most severe consequence if the gene had no canonical transcript. A variant was considered a high-confidence loss-of-function variant (stop-gained, splice site disrupting, or frameshift) if LOFTEE predicted it to be high-confidence without any warning flags. Missense variants were also annotated with MPC [18] and CADD [19] scores to assess likely pathogenicity, and were considered predicted damaging variants with an MPC score > 2 (for heterozygous variants) or CADD > 20 (for homozygous variants).

### Identifying likely pathogenic or pathogenic variants

To identify likely pathogenic or pathogenic variants in known ID genes, we downloaded (on March 18th 2022) a list of known developmental disorder genes from the DECIPHER database [2]. Variants were flagged as likely pathogenic if they were predicted to be a high-confidence loss-of-function or damaging missense variants in a DECIPHER gene with definitive or strong evidence and reported brain/cognition effects (*n* genes = 1142, listed in full in Supplementary Table 1), and sufficiently rare. Detailed information on rarity filters for variants can be found in the supplementary methods, and the full table of likely pathogenic variants is listed in Supplementary Table 2. All identification of likely pathogenic variants was performed using Hail [15] and executed in a Google Cloud Dataproc cluster.

### Rare variant association analysis

To assess the burden of rare, damaging heterozygous variants in known monoallelic ID genes in our intellectual disability cases, we further filtered likely pathogenic variants to those unreported (allele count = 0) in the Gnomad v2 [17] variant database. We then used a binomial logistic regression with participant sex and first ten principal components of genetic information as covariates. Similarly, to assess the burden of rare, damaging homozygous variants in biallelic ID genes we further filtered likely pathogenic variants to those unreported (n homozygotes = 0) in the Gnomad v2 variant database, and used Firth bias-corrected logistic regression with participant sex and first ten principal components as covariates. This analysis was repeated in male and female cases separately, with first ten principal components as covariates. For all rare variant association analyses, we removed NFID cases with clinically ambiguous karyotyping results (not clearly classified as benign or inherited from an unaffected parent).

For comparing the burden of rare, damaging heterozygous variants between case diagnostic subsets, we further filtered variants to those absent (allele count = 0) in both Gnomad [17] and all population control individuals. We then used a binomial logistic regression with participant sex, first ten principal components, and sequencing batch as covariates. Mild intellectual disability referred to individuals with ICD code F70, moderate, severe and profound ID referred to individuals with ICD codes F71-F73, and unspecified ID or developmental disorders referred to individuals with ICD codes F78-F79 and F80-89. Individuals were defined as having a relative with intellectual disability or a learning disability if they had either a relative enrolled as a case in the NFID study or were reported to have a relative with ID or a learning disability in patient questionnaires. Diagnosis of different forms of psychosis, including schizophrenia, were obtained from health care records and multi-professional evaluation. Sensory disabilities, learning disabilities, and dysmorphisms were binarized from free-text answers reported by physicians or care team members in study questionnaires. Full lists of sensory disabilities, learning disabilities, and dysmorphisms are reported in Supplementary Table 3. Bonferroni multiple testing correction for fourteen diagnostic phenotype comparisons was used to correct logistic regression results. To compare carrier rates in cases to controls, we additionally ascertained the rate of variants that were found in controls but absent in Gnomad and cases. Comparing the burden of rare, damaging homozygous variants between case diagnostic subsets was assessed with Firth bias-corrected logistic regression with participant sex, first ten principal components, and sequencing batch as covariates.

### De novo variant analysis

We assessed de novo variant status in trios using the Hail implementation of Samocha et al’s [20] de novo caller, which assesses the likelihood of putative de novo variants based on the proband and parental genotype qualities and reported reference population allele frequencies. All trios were confirmed by assessing kinship between parents and probands, and trios with incorrectly assigned parental relationships were removed from the analysis. We used for the population allele frequency prior either the reported Gnomad Finnish allele frequency or, if the variant was absent in Gnomad, the combined allele frequency of our samples and Gnomad (in-sample allele count – 1 / in-sample allele number + Gnomad Finnish allele number). Gnomad variants failing Gnomad internal quality control or with significantly different allele frequencies between Gnomad exomes and Gnomad genomes were not considered when choosing population allele frequency prior.

We then compared the observed number of high-confidence de novo variants to that expected by local mutation rates in DECIPHER genes with definitive or strong evidence, reported monoallelic, X heterozygous, or X hemizygous (males only) inheritance, and brain or cognitive effects. Baseline mutation rates [20] for synonymous, missense, and loss-of-function mutations at each gene were summed and multiplied by the number of cases (and by 2 haplotypes) to obtain the expected number of mutations in DECIPHER genes. We assessed the probability of observing ≥ N de novo mutations in the set of genes by calculating a Poisson *p*-value for the number of expected and observed de novo mutations. To account for differences in exome sequencing capture, we filtered to only trios where the proband and parents were sequenced with the same capture kit (*n* = 417 ID cases, *n* = 96 unaffected siblings), calculated the Poisson *p*-value separately in the set of trios captured with that kit, and combined the results with Fisher’s sum of logs method using the metap package [21] in R. This analysis was repeated in male and female cases only.

To assess differences in the rate of de novo mutations between case diagnostic subsets, we used binomial logistic regression with participant sex, first ten principal components, and sequencing batch as covariates. For this analysis, all trios with confirmed kinships were used, including those with mismatching parental and proband exome capture kits (*n* = 450) as this is accounted for by including sequencing batch as a covariate. Bonferroni multiple testing correction for fourteen diagnostic phenotype comparisons was used to correct logistic regression results.

### DNA array data processing and quality control

NFID samples were genotyped in seven batches on the Illumina Infinium CoreExome or Global Screening Array DNA microarray chips. FINRISK population controls were genotyped on the Illumina Infinium CoreExome, Global Screening Array, Human610-Quad, OmniExpress, or Affymetrix GeneChip chips. Health2000–2011 controls were genotyped in three batches on the Illumina Infinium CoreExome, Human610-Quad, or G4L chips. Samples were imputed using the Sequencing Initiative Suomi (SISU) (www.sisuproject.fi) v3 imputation panel containing 3775 high coverage (25–30x) whole genome sequences of Finnish individuals. Detailed information on DNA array data quality control and imputation can be found in the Supplementary Methods.

### Polygenic scores

To calculate polygenic scores, we downloaded genome-wide association study (GWAS) summary statistics for schizophrenia [22], ADHD [23], bipolar disorder [24], education attainment [25], focal epilepsy [26], generalized epilepsy [26], cognitive performance [25], major depression [27], intelligence [28], PTSD [29], and neuroticism [30]. We then used PRS-CS-auto [31] with a Finnish-specific linkage disequilibrium (LD) panel based on the SISu v3 imputation panel to calculate adjusted effect sizes conditional LD patterns in the reference data. We then LD pruned the imputed genotype data, calculated scores with Plink 1.9’s [32] score function and normalized the resultant scores to the control mean for each phenotype. To account for population stratification, we fit a linear model for the normalized scores including case status, sex, and first ten principal components as parameters. We then obtained the marginal mean and 95% confidence intervals for case status from the linear model using the emmeans [33] package in R.

### Rare and common variant interaction and multi-model inference

To assess whether the contribution of rare and common variants to ID is additive, we used a series of binomial logistic regressions for case status. For each regression, we included as parameters rare variant carrier status (whether the individual was a carrier of a LOF or missense MPC > 2 variant in a DECIPHER gene), polygenic score, and an interaction term between rare variant carrier status and polygenic score (in addition to sex and first ten principal components as covariates). Logistic regression results were corrected with Bonferroni multiple-testing correction for 11 polygenic score and rare variant interaction models.

To assess whether rare variants, common variants, or both best explained intellectual disability status, we used the MuMIn [34] package in R to calculate the Akaike Information Criterion (AIC) and Bayesian Information Criterion (BIC) between logistic regression models associating intellectual disability status to (1) only covariates (sex and ten first principal components), (2) covariates and polygenic score, (3) covariates and rare variant carrier status, or (4) covariates, polygenic score, and rare variant carrier status. For this analysis we chose to include only the cognitive performance polygenic score, as this PGS had the highest AIC and BIC explaining intellectual disability status when rare variant status and covariates were fixed (Supplementary Fig. 1).

## Results

### Rare and de novo variants are enriched in severe ID

**Fig. 1:**
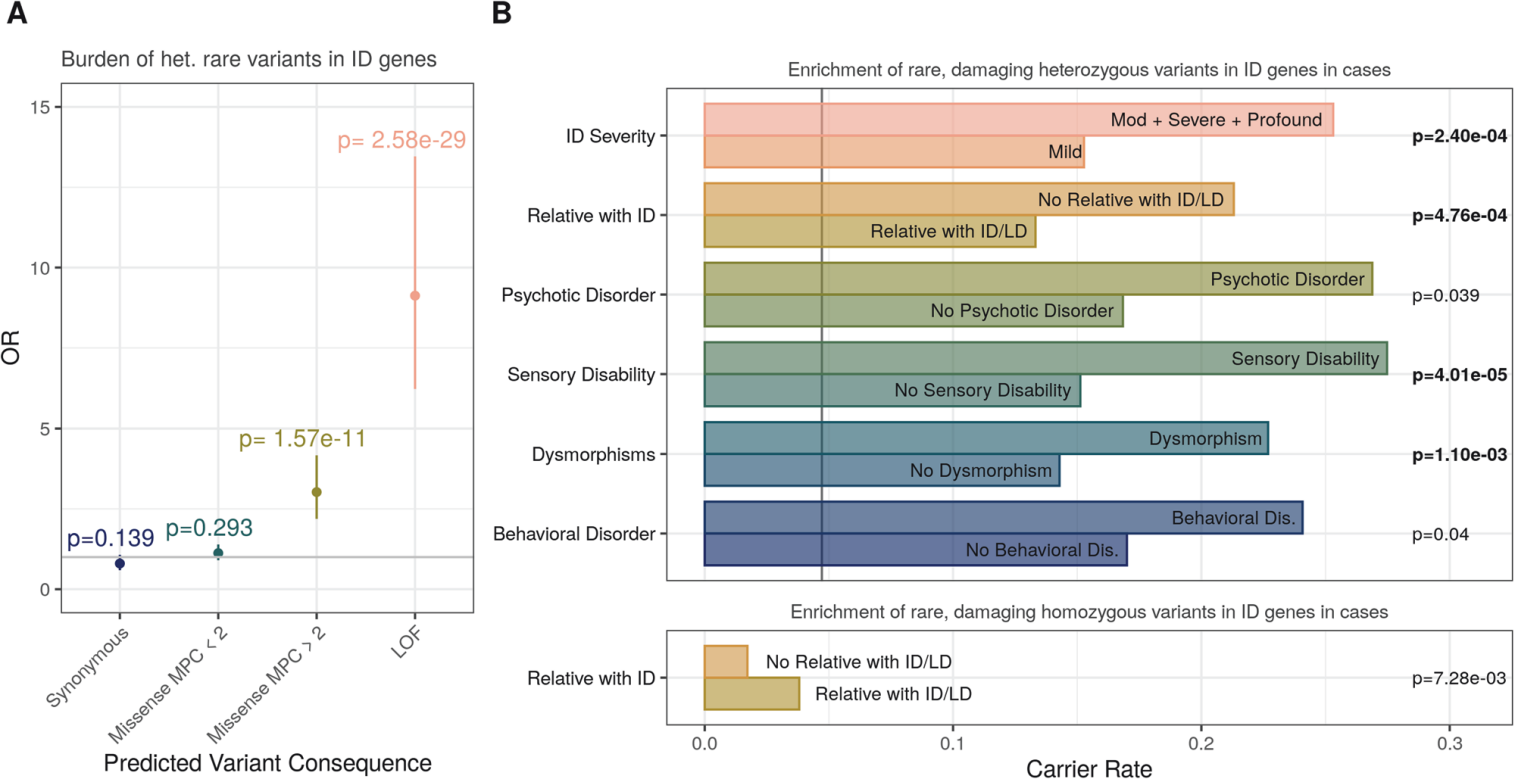
Burden of rare, damaging variants in known ID genes. **A** Burden of rare, damaging heterozygous variants in known monoallelic ID genes in NFID cases (*n* = 1055) compared to population controls (*n* = 4791). **B** Burden of rare, damaging variants in diagnostic case subsets. Bolded *p* values are those that pass Bonferroni multiple testing correction for 12 diagnostic phenotypes comparisons. Vertical grey line in heterozygous variant burden panel represents carrier rate of rare, LOF of damaging missense variants in ID genes found in controls, but not cases or Gnomad (0.047). There were no homozygous variants in recessive ID genes found in controls but not cases. Odds ratios and number of individuals for each case subset are found in Supplementary Table 4.

We found a significant burden of rare, damaging heterozygous variants in known dominant ID genes (Fig. 1A) and rare, damaging homozygous variants in known recessive ID genes (Supplementary Fig. 2) in NFID patients compared to population controls, in line with previous analysis with the NFID cohort [6]. This burden was similar in males and females when examined separately (Supplementary Fig. 16). When we compared the relative burden of rare variants in ID case subsets, we found a significant burden of rare, damaging heterozygous variants in patients with more severe ID, a relative with ID or a learning disability, a sensory disability (e.g. hearing or vision impairment), or dysmorphic features (Fig. 1B). We also found a nominally significant burden of rare, damaging variants in ID patients with a behavioral or psychotic disorder. To account for the increased burden of severe ID in patients with sensory disabilities, dysmorphic features, and without a relative with ID (Supplementary Fig. 3), we adjusted our logistic regression by including level of ID as a covariate when testing for an excess of rare variants. We found a significant burden in these three comorbidity classes even after adjusting for level of intellectual disability (Supplementary Fig. 4). In contrast, the only case subset with a burden of rare, damaging homozygous variants in known recessive ID genes were individuals with a reported relative with ID or a learning disability, the opposite burden for rare variants in dominant ID genes. In most case subsets, the proportion of males and females was not significantly different (Supplementary Fig. 18).

**Fig. 2:**
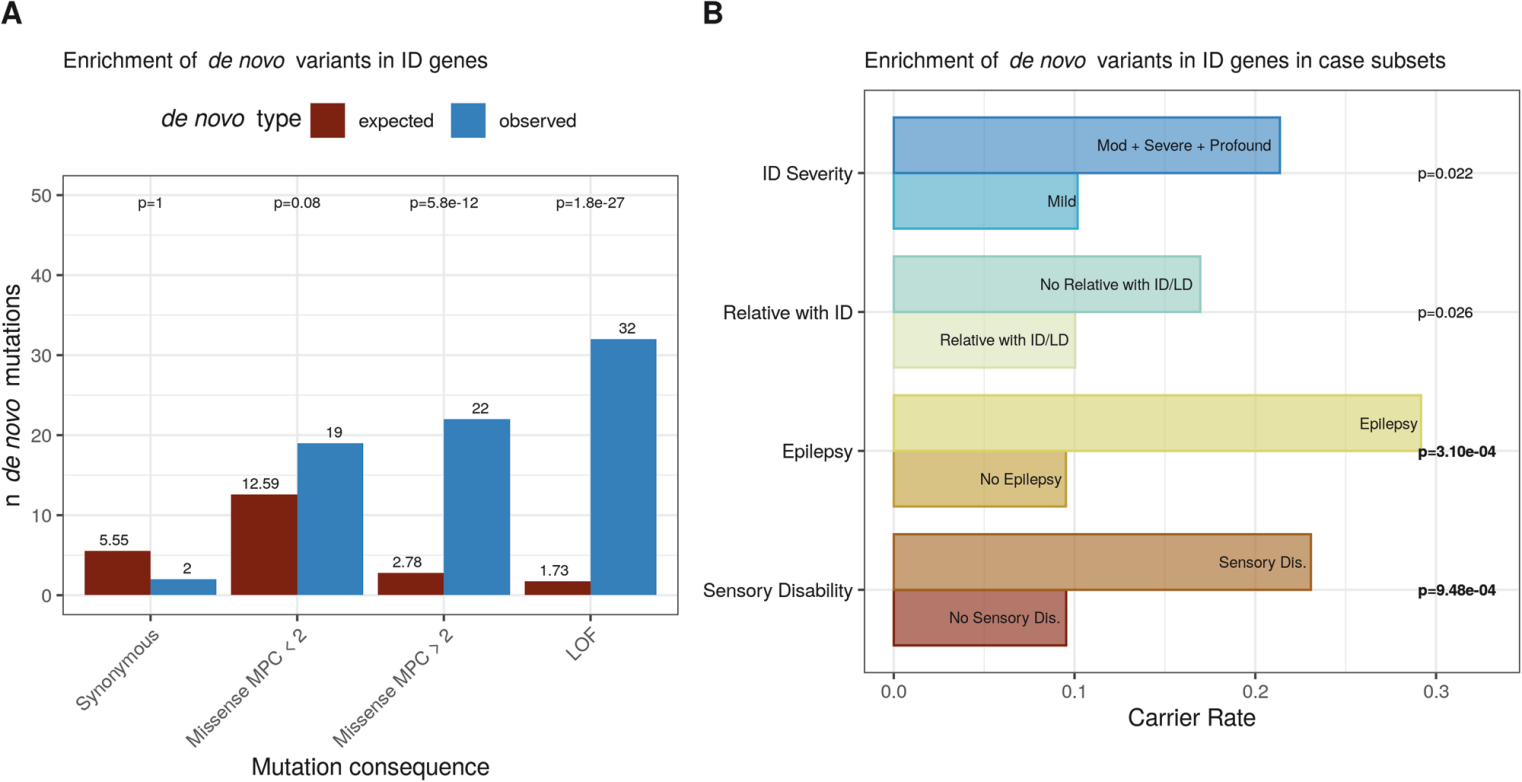
Enrichment of de novo variants in known ID genes. **A** Comparison of observed vs. expected de novo variants in known ID genes in ID cases (*n* = 417). **B** Enrichment of de novo variants in ID genes in case diagnostic subsets (*n* = 450). Bolded *p* values are those that pass Bonferroni multiple testing correction for 12 diagnostic phenotypes. Odds ratios and number of individuals for each case subset are found in Supplementary Table 5.

We also found significantly more de novo damaging variants in known dominant ID genes than expected by baseline mutation rates in NFID cases (Fig. 2A), and no difference in unaffected siblings (Supplementary Fig. 5). This burden of de novo damaging variation was significant in both males and females. We saw a significant enrichment of de novo damaging variants in dominant ID genes in individuals with epilepsy or a sensory disability, and a nominally significant burden in individuals with more severe ID and without a relative with ID or a learning disability (Fig. 2B). Considering the substantial overlap between genes associated with both cognitive impairment, epilepsy, and sensory disabilities (Supplementary Fig. 15), we believe this enrichment reflects a more syndromic presentation of intellectual disability rather than epilepsy or sensory disabilites leading to later cognitive impairment. Additionally, after accounting for the increased burden of severe ID in the other three case comorbiditites, we found that the burden of de novo variants in individuals with epilepsy or a sensory disability remained significant (Supplementary Fig. 6).

### Common variants are enriched in both mild and severe ID

We found a significant difference between ID cases and population controls in polygenic scores for ADHD, intelligence, cognitive performance, and educational attainment, as well as nominally significant differences in polygenic scores for schizophrenia and major depression that were no longer significant after multiple testing correction (Supplementary Fig. 7). When comparing the common variant burden between mild and more severe ID, we saw a trend for lower polygenic score for educational attainment in mild ID (Supplementary Fig. 8) that was not statistically significant. Similarly, we saw a trend for lower polygenic scores for educational attainment, cognitive performance, and intelligence in individuals with a relative with ID compared to those without (Supplementary Fig. 9) and a trend for lower polygenic scores for intelligence and cognitive performance in individuals who were not carriers of a rare variant (Supplementary Fig. 10). None of these trends were found to be statistically significant, however.

There were several comparisons with nominally significant differences in polygenic scores when comparing between case diagnostic subsets, but none of these findings passed multiple testing correction (Supplementary Fig. 11).

### Rare and common variants are additive throughout the spectrum of ID

**Fig. 3:**
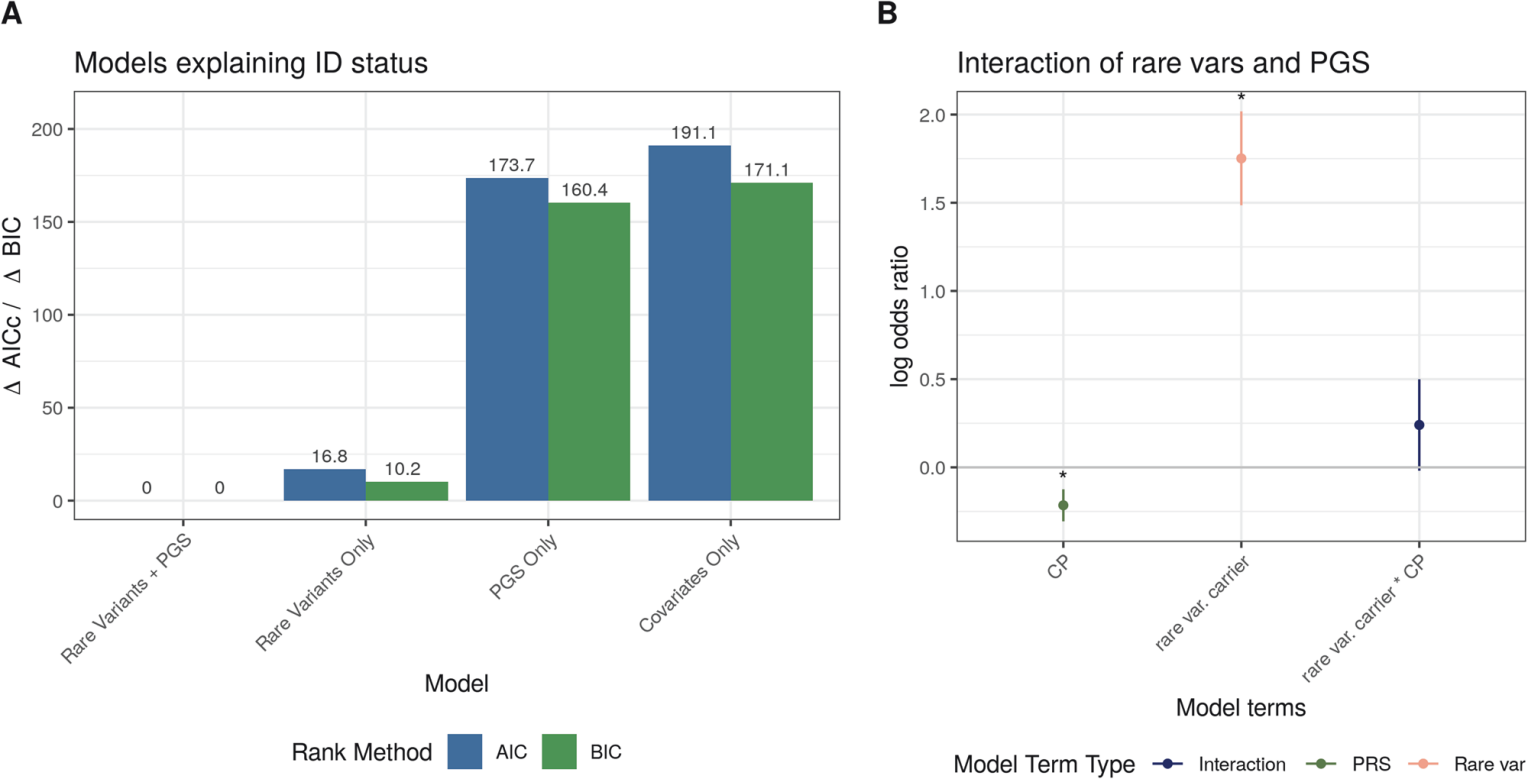
Rare and common variant additivity in ID cases. **A** Comparison of logistic regression models predicting intellectual disability status, where the model with the lowest ΔAICc or ΔBIC is the model that best explains the data. Covariates Only: sex and first ten principal components, PGS Only: cognitive performance PGS and covariates, Rare Variants Only: LOF and damaging missense carrier status and covariates, Rare Variants + PGS: LOF and damaging missense carrier status, cognitive performance PGS, and covariates. **B** Logistic regression comparing ID cases to population controls, including both LOF or missense MPC > 2 variant carrier status, polygenic score, and an interaction term between rare variant carrier status and polygenic score in the model. CP cognitive performance, *indicates Bonferroni-adjusted *p* < 0.05.

We also examined the interaction between polygenic burden and the presence of rare, damaging variants in known ID genes. For loss-of-function and damaging missense variants, we observed no interaction between rare variant carrier status and polygenic risk in intelligence, cognitive performance, or educational attainment contributing to intellectual disability (Fig. 3B, Supplementary Fig. 12). In other words, the contribution of polygenic variants affecting cognitive traits and rare, damaging variants in known ID genes were additive, not multiplicative. We also examined the interaction between polygenic burden and rare variant burden in specific case sets. In individuals with mild ID or a relative with ID or a learning disability, we saw both a significant burden of LOF or damaging missense variants and a significant effect of common variants associated with intelligence, educational attainment, cognitive performance, and ADHD with little evidence of an interaction between the two (Supplementary Fig. 13). In individuals with more severe ID and without a relative with ID or a learning disability, we saw a significant burden of rare, damaging variants but an attenuated burden of common variants, likely due to lack of power (Supplementary Fig. 14).

To further explore the contribution of common and rare variants to ID, we used a multi-model inference approach to evaluate which models best explain intellectual disability status: a model including both polygenic score and rare variant carrier status as exposure variables, models including either polygenic burden or rare variant carrier status as the exposure variable, or fixed covariates (sex, first ten principal components of genetic variation) only. We found that the model containing both rare and common variants best explained the data, with notable differences in explanatory power in both the Akaike Information Criterion and Bayesian Information Criterion (Fig. 3A).

### Recessive ID caused by Finnish-enriched variants is rare

**Fig. 4:**
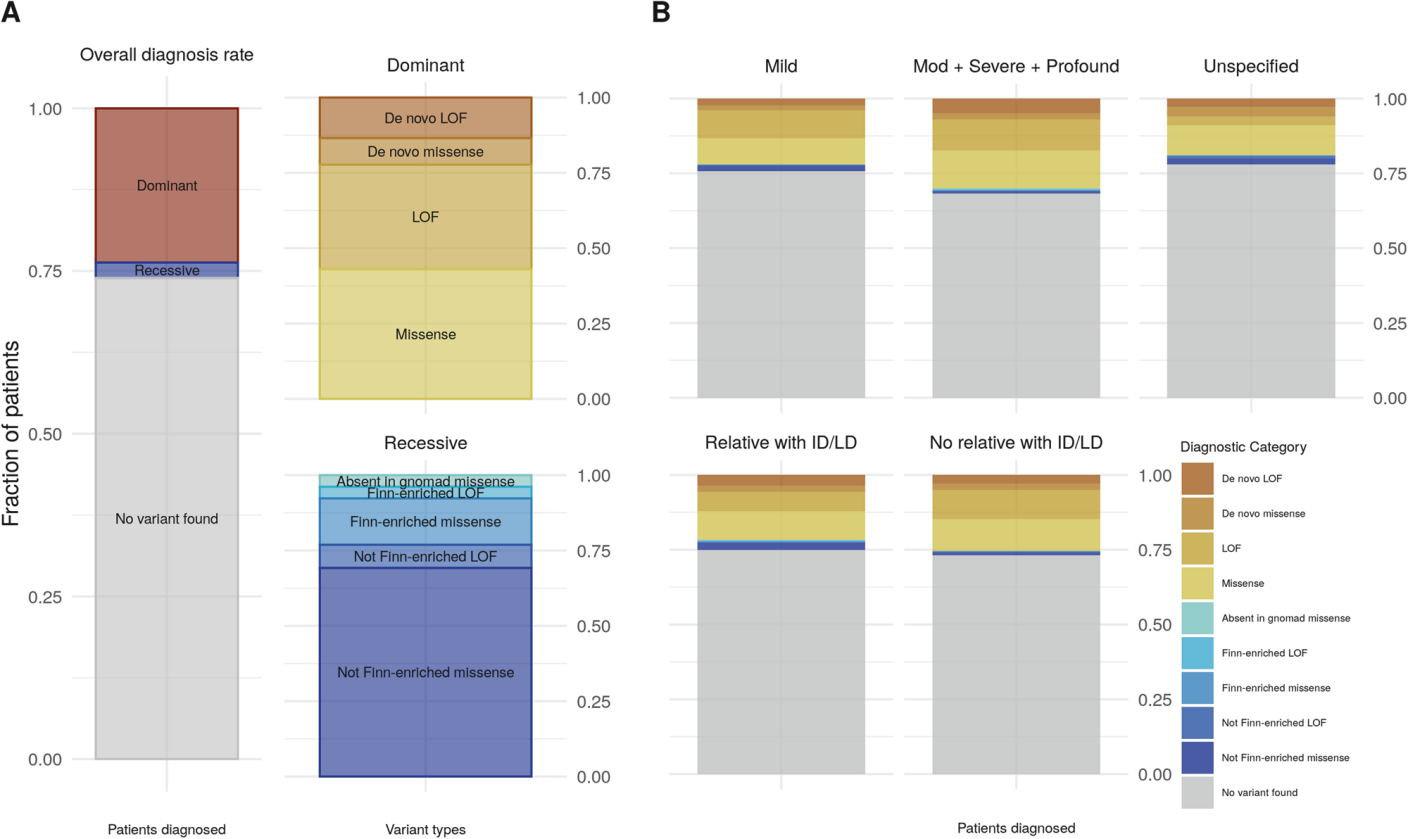
Diagnostic rate in ID cases. **A** Overall diagnostic rate for ID patients (25.62%, *n* = 281), of which 23.25% (*n* = 255) were found to have a heterozygous variant in a known dominant ID gene and 2.37% (*n* = 26) were found to have a homozygous variant in known recessive ID gene. **B** Diagnostic rate for patient subsets. The total diagnostic rate for moderate, severe, and profound ID (31.52%, *n* = 104) was higher than that of mild and unspecified ID (24.07%, *n* = 138). The total diagnostic rate for individuals without a family member with ID or a learning disability (26.7%, *n* = 160) was higher than that for individuals with a family member with ID/LD (24.31%, *n* = 121).

We found that 23.25% of individuals (*n* = 255) carried a rare, pathogenic or likely pathogenic heterozygous variant in known dominant ID genes, while 2.37% (*n* = 26) carried a rare damaging homozygous variant in known recessive ID genes, for a total diagnostic rate of 25.62% (Fig. 4a). For dominant variants, 47.8% (*n* = 122) were high-confidence LOF variants (de novo LOF *n* = 32) while 52.2% (*n* = 133) were damaging missense variants (de novo damaging missense *n* = 22). For recessive variants, we found 11.5% (*n* = 3) were high-confidence LOF variants (Finnish-enriched *n* = 1) while 88.5% (*n* = 23) were damaging missense (Finnish-enriched *n* = 5).

Variants were considered Finnish-enriched if the allele frequency in the Gnomad v2 Finnish population was at least twofold greater than any other population, or the variant was absent in Gnomad v2 (i.e., present only in the Finnish population). The diagnostic rate and composition of dominant and recessive variants here reflects the makeup of the Northern Finnish Intellectual Disability cohort, which has been specifically ascertained for individuals *without* a known genetic or environmental cause for their ID, and may not reflect the actual population prevalence. In particular, known Finnish disease heritage genes are likely routinely screened in the clinic and individuals with damaging variants in these genes would not be eligible for enrollment in the NFID study. However, despite this caveat and the known enrichment of rare, damaging variants in Finland, recessive ID due to Finnish-enriched variants explains the etiology of only a small percentage of patients (*n* = 6, 0.5%) in our study.

The exome diagnostic rate for moderate, severe, and profound ID (31.52%, *n* = 104) was higher than that of mild and unspecified ID (24.07%, *n* = 138), and the diagnostic rate for individuals without a family member with ID or a learning disability (26.7%, *n* = 160) was higher than that for individuals with a family member with ID/LD (24.31%, *n* = 121) (Fig. 4B). Of the participants with a rare, damaging variant, there were relatively more dominant variants in moderate, severe, and profound ID (94.2%, *n* = 98) than in mild ID (87.7%, *n* = 121) and in individuals without a relative with ID/LD (94.4%, *n* = 151) than in those with a relative with ID (86.0%, *n* = 104).

## Discussion

Finland is known to have an increase of low-frequency damaging variants [9] and an internal bottleneck in the northern part of the country [10]. In our cohort recruited from Northern Finland, we found a burden of rare, damaging homozygous variants in known recessive ID genes, reflecting the presence of autosomal recessive ID in families with multiple affected probands. Yet overall, these homozygous variants explain only a small proportion of ID cases in our cohort (both familial and sporadic), pointing to the fact that autosomal recessive ID is still a relatively uncommon cause for ID in the Finnish population [35].

A larger proportion of ID in our study was explained by dominant variation. We found a significant burden of rare loss of function and damaging missense variants in known ID genes in all patients, both mild and severe. This burden was enriched in ID cases with more severe cognitive impairment and with features typically associated with monogenic intellectual disability: dysmorphic features or a sensory disability such as hearing or vision impairment. Similarly, the burden of damaging de novo variants in known ID genes was enriched in individuals with a sensory disability or epilepsy, again features usually associated with monogenic ID. Patients with more severe ID and monogenic disease-associated features in our cohort are then more likely to have a rare, damaging dominant variant than mild ID patients, but there is still a burden of these rare, damaging variants in mild ID patients compared to normal IQ individuals. This burden of rare variation in mild ID patients may appear to be counterintuitive considering the evidence that factors influencing mild ID are similar to those influencing normal cognitive functioning, but recent work has shown that rare, damaging variants contribute to reduced cognitive functioning among even normal IQ individuals [3, 4]. We therefore suggest that exome sequencing, including the analysis of potential rare damaging variants, should also be considered as a first-tier diagnostic test for patients with mild ID.

In addition to a burden of rare, damaging variation, we found a significant common variant burden in mild ID, severe ID and other patients with features typically associated with monogenic ID. We were unable to detect a difference in the common variant burden between mild and more severely affected individuals or patients with monogenic ID-associated features, though in our cohort this may be due to lack of power. This is in line with previous work that found that common genetic variation affects both the overall risk and clinical presentation in severe neurodevelopmental disorders previously considered to be monogenic [5, 6], even those in families targeted for rare monogenic variant discovery [36].

When we included both common and rare variants in the same model, we found that rare and common variant burdens were additive throughout the spectrum of ID severity. Common and rare variants together also had a notably better power in explaining ID case status than rare or common variation alone. This evidence for the additivity of rare and common variants in ID in our cohort, throughout the spectrum of cognitive impairment, family pedigree type, and comorbidity with monogenic disease-associated features, suggests that there is an overlap of genetic risk factors that give complicated pedigrees of individuals with varying levels of affectedness. This model of overlapping genetic risk factors has recently been proposed in autism spectrum disorders [37].

Overall, we report here evidence for the additive burden of rare, damaging variants and common variants affecting cognitive traits throughout the spectrum of mild to more severe ID. Further research into the specific characteristics of variants and genes and their combination in individuals and families is needed to fully explain the genetic factors contributing to the wide spectrum of affectedness in intellectual disability.

### Supplementary information

Supplementary Figures and Methods

Supplementary Table 1

Supplementary Table 2

## Data Availability

All summary level data are available from the corresponding author on reasonable request. The datasets generated and/or analyzed during the current study are not publicly available due to patient confidentiality. Data from the FINRISK and Health2000– 2011 studies are available on application to THL Biobank (www.thl.fi/en/web/thl-biobank). The data from the Northern Finnish Intellectual Disability study are available from the corresponding author upon reasonable request.

## Code availability

All code used for analyses presented in the manuscript is available from the corresponding author upon reasonable request. The exome sequencing data quality control pipeline is available at in a public repository at https://github.com/lea-urpa/exome_qc_library.

## References

[1] Maulik PK, Mascarenhas MN, Mathers CD, Dua T, Saxena S (2011). Prevalence of intellectual disability: a meta-analysis of population-based studies. Res Dev Disabil.

[2] Firth HV, Richards SM, Bevan AP, Clayton S, Corpas M, Rajan D (2009). DECIPHER: database of chromosomal imbalance and phenotype in humans using ensembl resources. Am J Hum Genet.

[3] Reichenberg A, Cederlöf M, McMillan A, Trzaskowski M, Kapra O, Fruchter E (2016). Discontinuity in the genetic and environmental causes of the intellectual disability spectrum. Proc Natl Acad Sci USA.

[4] Lichtenstein P, Tideman M, Sullivan PF, Serlachius E, Larsson H, Kuja-Halkola R (2022). Familial risk and heritability of intellectual disability: a population-based cohort study in Sweden. J Child Psychol Psychiatry.

[5] Niemi MEK, Martin HC, Rice DL, Gallone G, Gordon S, Kelemen M (2018). Common genetic variants contribute to risk of rare severe neurodevelopmental disorders. Nature.

[6] Kurki MI, Saarentaus E, Pietiläinen O, Gormley P, Lal D, Kerminen S (2019). Contribution of rare and common variants to intellectual disability in a sub-isolate of Northern Finland. Nat Commun.

[7] Chen CY, Tian R, Ge T, Lam M, Sanchez-Andrade G, Singh T, et al. The impact of rare protein coding genetic variation on adult cognitive function [Internet]. medRxiv; 2022. p. 2022.06.24.22276728. Available from: https://www.medrxiv.org/content/10.1101/2022.06.24.22276728v1 10.1038/s41588-023-01398-8 PMC10260403 37231097

[8] Kingdom R, Tuke M, Wood A, Beaumont RN, Frayling TM, Weedon MN (2022). Rare genetic variants in genes and loci linked to dominant monogenic developmental disorders cause milder related phenotypes in the general population. Am J Hum Genet.

[9] Lim ET, Würtz P, Havulinna AS, Palta P, Tukiainen T, Rehnström K (2014). Distribution and medical impact of loss-of-function variants in the Finnish founder population. PLOS Genet.

[10] Norio R (2003). Finnish disease heritage I. Hum Genet.

[11] Vartiainen E, Laatikainen T, Peltonen M, Juolevi A, Männistö S, Sundvall J (2010). Thirty-five-year trends in cardiovascular risk factors in Finland. Int J Epidemiol.

[12] Gould R, Ilmarinen J, Järvisalo J, Koskinen S. Dimensions of work ability: results of the health 2000 survey. 2022. Available from: https://www.julkari.fi/handle/10024/78055.

[13] Rivas MA, Graham D, Sulem P, Stevens C, Desch AN, Goyette P (2016). A protein-truncating R179X variant in RNF186 confers protection against ulcerative colitis. Nat Commun.

[14] Robust relationship inference in genome-wide association studies | Bioinformatics | Oxford Academic. (2022). Available from: https://academic.oup.com/bioinformatics/article/26/22/2867/228512 10.1093/bioinformatics/btq559 PMC3025716 20926424

[15] Hail Team. Hail https://github.com/hail-is/hail/commit/39909e0a396f. Available from: https://github.com/hail-is/hail/commit/39909e0a396f.

[16] McLaren W, Gil L, Hunt SE, Riat HS, Ritchie GRS, Thormann A (2016). The ensembl variant effect predictor. Genome Biol.

[17] Karczewski KJ, Francioli LC, Tiao G, Cummings BB, Alföldi J, Wang Q (2020). The mutational constraint spectrum quantified from variation in 141,456 humans. Nature.

[18] Samocha KE, Kosmicki JA, Karczewski KJ, O’Donnell-Luria AH, Pierce-Hoffman E, MacArthur DG, et al. Regional missense constraint improves variant deleteriousness prediction. bioRxiv; 2017. p. 148353. Available from: https://www.biorxiv.org/content/10.1101/148353v1

[19] Rentzsch P, Witten D, Cooper GM, Shendure J, Kircher M (2019). CADD: predicting the deleteriousness of variants throughout the human genome. Nucleic Acids Res.

[20] Samocha KE, Robinson EB, Sanders SJ, Stevens C, Sabo A, McGrath LM (2014). A framework for the interpretation of de novo mutation in human disease. Nat Genet.

[21] Dewey M. metap: meta-analysis of significance values. 2022. Available from: https://CRAN.R-project.org/package=metap

[22] Consortium TSWG of the PG, Ripke S, Walters JT, O’Donovan MC. Mapping genomic loci prioritises genes and implicates synaptic biology in schizophrenia. medRxiv; 2020. p. 2020.09.12.20192922. Available from: https://www.medrxiv.org/content/10.1101/2020.09.12.20192922v1

[23] Demontis D, Walters RK, Martin J, Mattheisen M, Als TD, Agerbo E, et al. Discovery of the first genome-wide significant risk loci for ADHD. bioRxiv; 2017. p. 145581. Available from: https://www.biorxiv.org/content/10.1101/145581v1 10.1038/s41588-018-0269-7 PMC6481311 30478444

[24] Stahl EA, Breen G, Forstner AJ, McQuillin A, Ripke S, Trubetskoy V (2019). Genome-wide association study identifies 30 Loci Associated with Bipolar Disorder. Nat Genet.

[25] Lee JJ, Wedow R, Okbay A, Kong E, Maghzian O, Zacher M (2018). Gene discovery and polygenic prediction from a genome-wide association study of educational attainment in 1.1 million individuals. Nat Genet.

[26] Abou-Khalil B, Auce P, Avbersek A, Bahlo M, Balding DJ, Bast T (2018). Genome-wide mega-analysis identifies 16 loci and highlights diverse biological mechanisms in the common epilepsies. Nat Commun.

[27] Wray NR, Ripke S, Mattheisen M, Trzaskowski M, Byrne EM, Abdellaoui A (2018). Genome-wide association analyses identify 44 risk variants and refine the genetic architecture of major depression. Nat Genet.

[28] Savage JE, Jansen PR, Stringer S, Watanabe K, Bryois J, de Leeuw CA (2018). Genome-wide association meta-analysis in 269,867 individuals identifies new genetic and functional links to intelligence. Nat Genet.

[29] Duncan LE, Ratanatharathorn A, Aiello AE, Almli LM, Amstadter AB, Ashley-Koch AE (2018). Largest GWAS of PTSD (N=20 070) yields genetic overlap with schizophrenia and sex differences in heritability. Mol Psychiatry.

[30] Nagel M, Jansen PR, Stringer S, Watanabe K, de Leeuw CA, Bryois J (2018). Meta-analysis of genome-wide association studies for neuroticism in 449,484 individuals identifies novel genetic loci and pathways. Nat Genet.

[31] Ge T, Chen CY, Ni Y, Feng YCA, Smoller JW (2019). Polygenic prediction via Bayesian regression and continuous shrinkage priors. Nat Commun.

[32] Chang CC, Chow CC, Tellier LC, Vattikuti S, Purcell SM, Lee JJ (2015). Second-generation PLINK: rising to the challenge of larger and richer datasets. GigaScience.

[33] Lenth RV, Buerkner P, Herve M, Jung M, Love J, Miguez F, et al. Emmeans: estimated marginal means, aka least-squares means. Available from: https://cran.r-project.org/web/packages/emmeans/index.html

[34] Bartón K. MuMIn: Multi-model inference. 2022. Available from: https://cran.r-project.org/web/packages/MuMIn/MuMIn.pdf

[35] Järvelä I, Määttä T, Acharya A, Leppälä J, Jhangiani SN, Arvio M (2021). Exome sequencing reveals predominantly de novo variants in disorders with intellectual disability (ID) in the founder population of Finland. Hum Genet.

[36] Oliver KL, Ellis CA, Scheffer IE, Ganesan S, Leu C, Sadleir LG, et al. Common risk variants for epilepsy are enriched in families previously targeted for rare monogenic variant discovery. eBioMedicine. 2022;81. Available from: https://www.thelancet.com/journals/ebiom/article/PIIS2352-3964(22)00260-2/fulltext 10.1016/j.ebiom.2022.104079 PMC9156876 35636315

[37] Wang T, Zhao PA, Eichler EE (2022). Rare variants and the oligogenic architecture of autism. Trends Genet.

