# Supplementary material for: Evidence for the additivity of rare and common variant burden throughout the spectrum of intellectual disability
Source: Eur J Hum Genet. 2024 Mar 11;32(5):576–83. doi: 10.1038/s41431-024-01581-3 (PMC11061114; doi:10.1038/s41431-024-01581-3)
Supplement: Supplementary file 1 — Supplementary Figures and Methods [file 41431_2024_1581_MOESM1_ESM.docx]

Table of Contents

[Supplementary Figures and Tables 2](#_Toc156309850)

[Supplementary Figures 2](#_Toc156309851)

[Supplementary Figure 1. Comparison of models with intelligence-related polygenic scores. 2](#_Toc156309852)

[Supplementary Figure 2. Burden of rare, damaging homozygous variants in ID known genes. 2](#_Toc156309853)

[Supplementary Figure 3. Proportion of Mild ID cases in case subsets 3](#_Toc156309854)

[Supplementary Figure 4. Burden of rare heterozygous variants in ID diagnostic subsets, with ID level as covariate 3](#_Toc156309855)

[Supplementary Figure 5. Comparison between observed and expected de novo variants in unaffected relatives 4](#_Toc156309856)

[Supplementary Figure 6. Burden of de novo variants in ID diagnostic subsets, with ID level as covariate. 4](#_Toc156309857)

[Supplementary Figure 7. Polygenic burden ID cases compared to population controls 5](#_Toc156309858)

[Supplementary Figure 8. Polygenic burden in mild vs more severe ID cases 5](#_Toc156309859)

[Supplementary Figure 9. Polygenic burden in individuals with a relative with ID or learning disability vs those without. 6](#_Toc156309860)

[Supplementary Figure 10. Polygenic burden in rare variant carriers vs non-carriers 6](#_Toc156309861)

[Supplementary Figure 11. Nominally significant differences in polygenic score between diagnostic ID case subsets. 7](#_Toc156309862)

[Supplementary Figure 12. Interaction between rare variants in known ID genes and polygenic score. 8](#_Toc156309863)

[Supplementary Figure 13. Interaction between rare and common variants in cases with mild ID or a relative with ID 8](#_Toc156309864)

[Supplementary Figure 14. Interaction between rare and common variants in cases with severe ID or without a relative with ID. 9](#_Toc156309865)

[Supplementary Figure 15. Pleiotropy in high-confidence developmental disorder genes. 10](#_Toc156309866)

[Supplementary Figure 16. Rare variant burden in known ID genes in males and females. 10](#_Toc156309867)

[Supplementary Figure 17. Enrichment of *de novo* variants in known ID genes in males and females. 11](#_Toc156309868)

[Supplementary Figure 18. Proportion of male and female cases in diagnostic subsets. 11](#_Toc156309869)

[Supplementary Tables 12](#_Toc156309870)

[Supplementary Table 3. Patient phenotypes for binary categorical variables 12](#_Toc156309871)

[Supplementary Table 4. Burden of rare, damaging variants in known ID genes 13](#_Toc156309872)

[Supplementary Table 5. Enrichment of de novo variants in known ID genes 13](#_Toc156309873)

[Supplementary Methods 14](#_Toc156309874)

[Northern Finland Intellectual Disability cohort 14](#_Toc156309875)

[Population control individuals 14](#_Toc156309876)

[Exome sequencing and quality control 15](#_Toc156309877)

[Rarity filters for identifying likely pathogenic variants 16](#_Toc156309878)

[DNA array data processing and quality control 17](#_Toc156309879)

[Cognitive impairment pleiotropy 18](#_Toc156309880)

[References 19](#_Toc156309881)

# Supplementary Figures and Tables

## Supplementary Figures


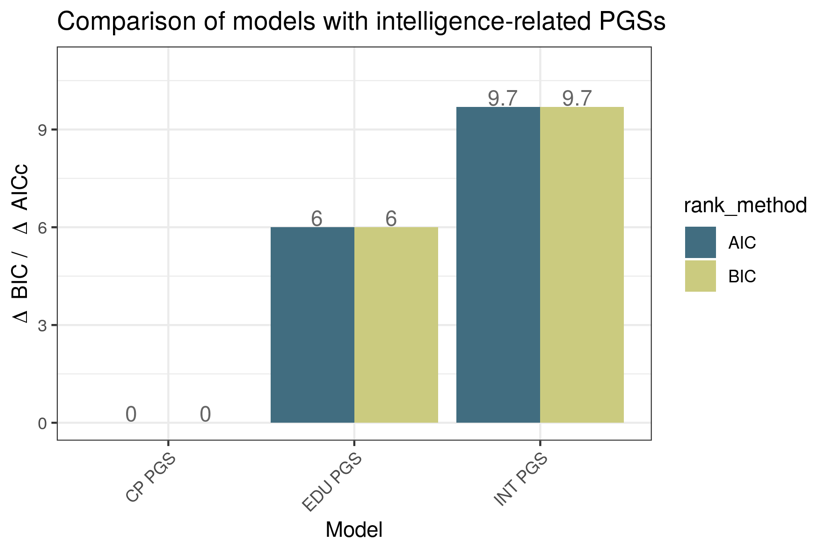


Supplementary Figure 1. Comparison of models with intelligence-related polygenic scores. Comparison of logistic regression models predicting intellectual disability case status, where LOF and damaging missense rare variant carrier status and covariates (sex, first ten principal components) are fixed and polygenic score was varied. CP = cognitive performance, EDU = educational attainment, INT = intelligence.


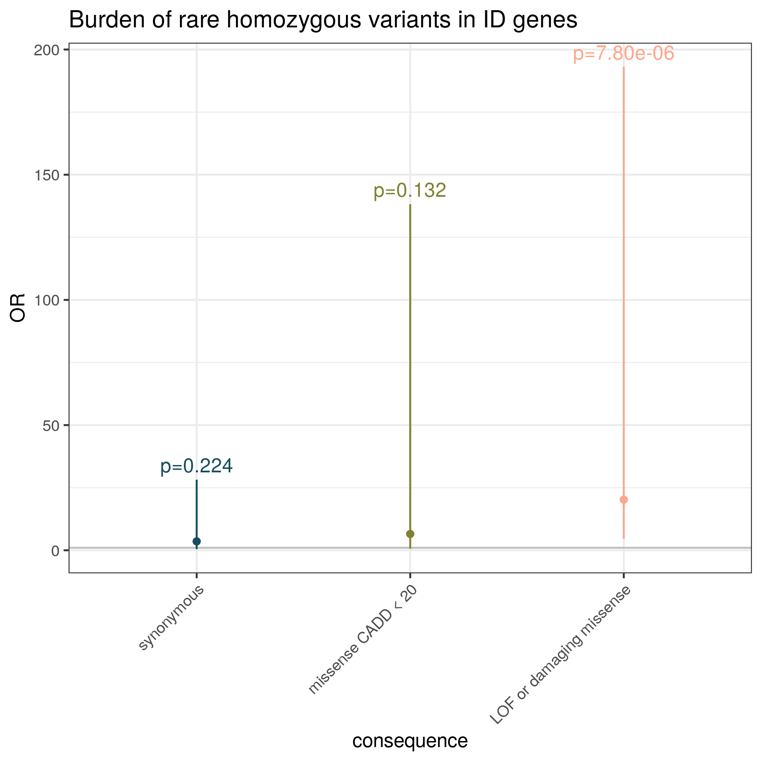


Supplementary Figure 2. Burden of rare, damaging homozygous variants in ID known genes. Burden of rare, damaging homozygous variants in known biallelic ID genes in NFID cases (n=1055) compared to population controls (n=4791). LOF or missense CADD > 20 OR=20.2 (4.53-192 95% CI, p=7.80e-6), missense CADD < 20 OR=6.52 (0.63-138 95% CI, p=0.132), synonymous OR=3.56 (0.44-28.2 95% CI, p=0.224).


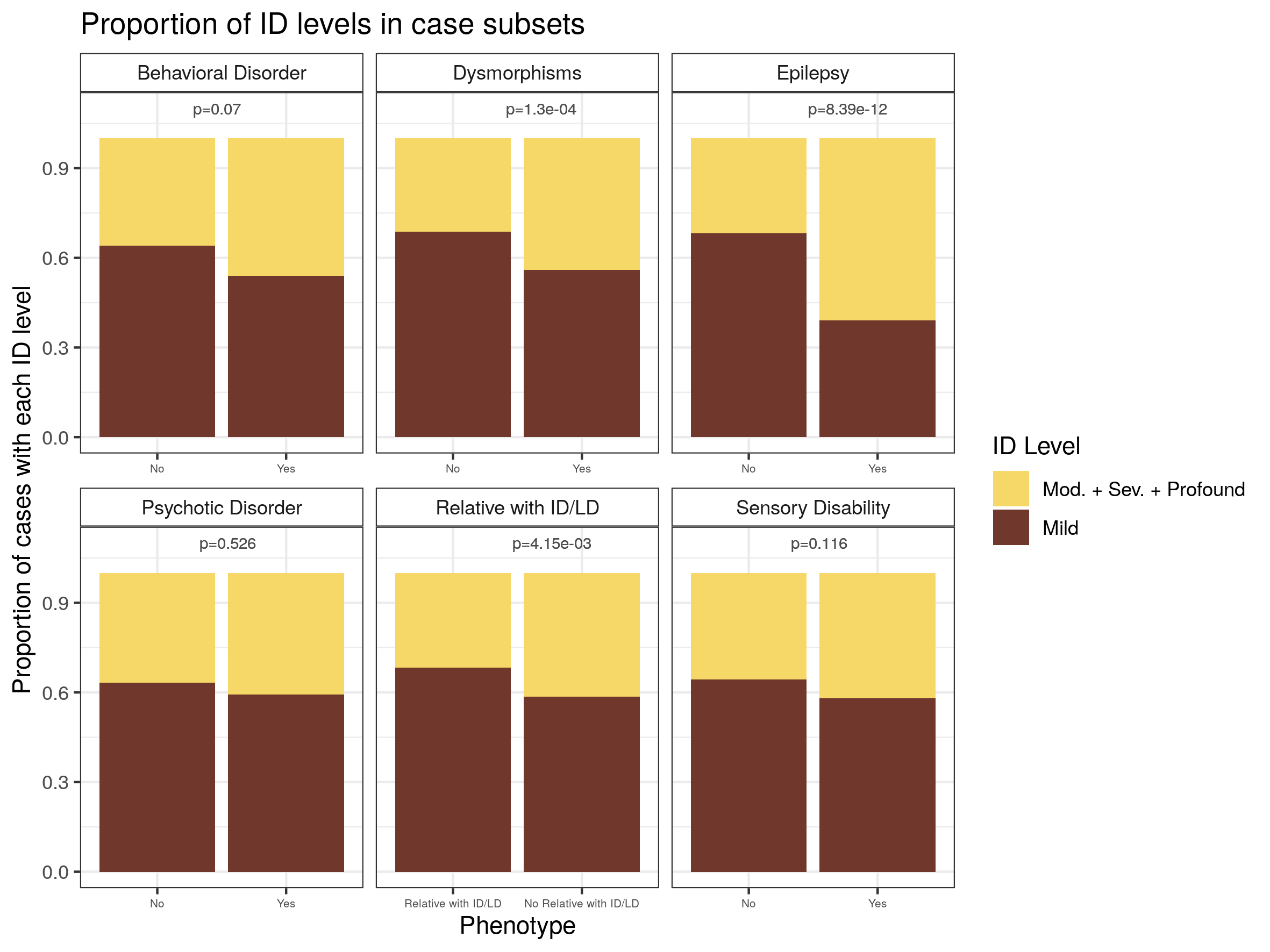


Supplementary Figure 3. Proportion of Mild ID cases in case subsets**.** Comparison of the proportion of mild and moderate + severe + profound ID cases among other cases subsets. P values represent Chi square test for difference of proportions in ID levels between diagnostic case comparisons.

**
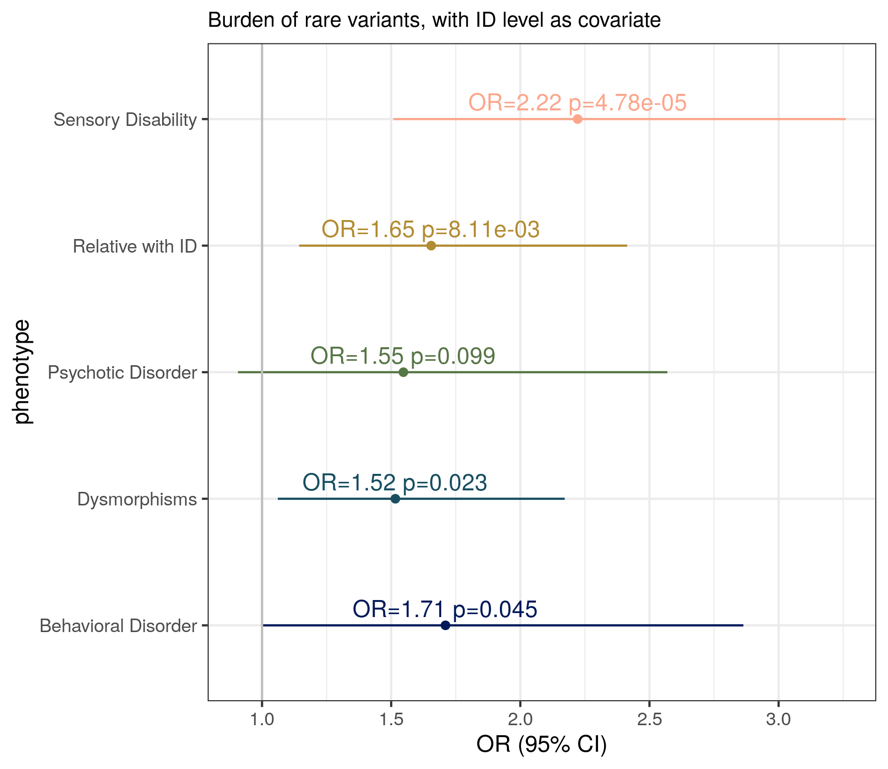
**

Supplementary Figure 4. Burden of rare heterozygous variants in ID diagnostic subsets, with ID level as covariate**.** Burden of rare variants in known ID genes in ID diagnostic subsets, including ID level as a covariate in the model.


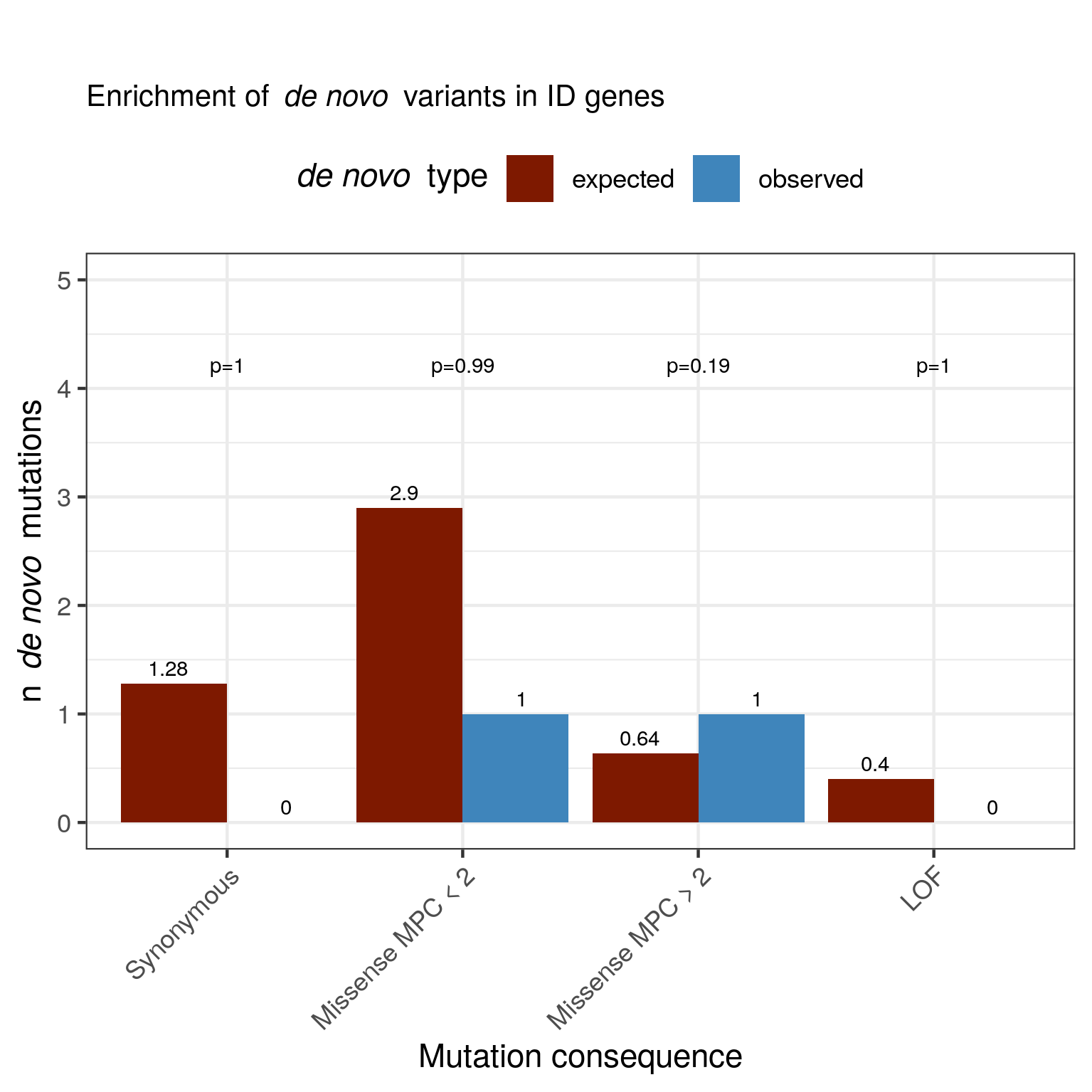


Supplementary Figure 5. Comparison between observed and expected de novo variants in unaffected relatives**.** Comparison of observed vs expected *de novo* variants in known ID genes in unaffected siblings of ID cases (n=96).


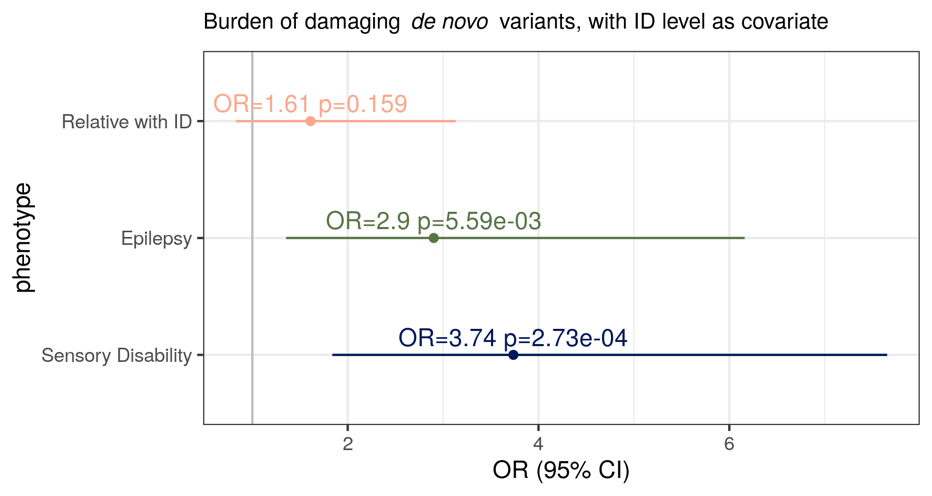


Supplementary Figure 6. Burden of de novo variants in ID diagnostic subsets, with ID level as covariate. Burden of *de novo* variants in known ID genes ID diagnostic subsets, including ID level as a covariate in the model.


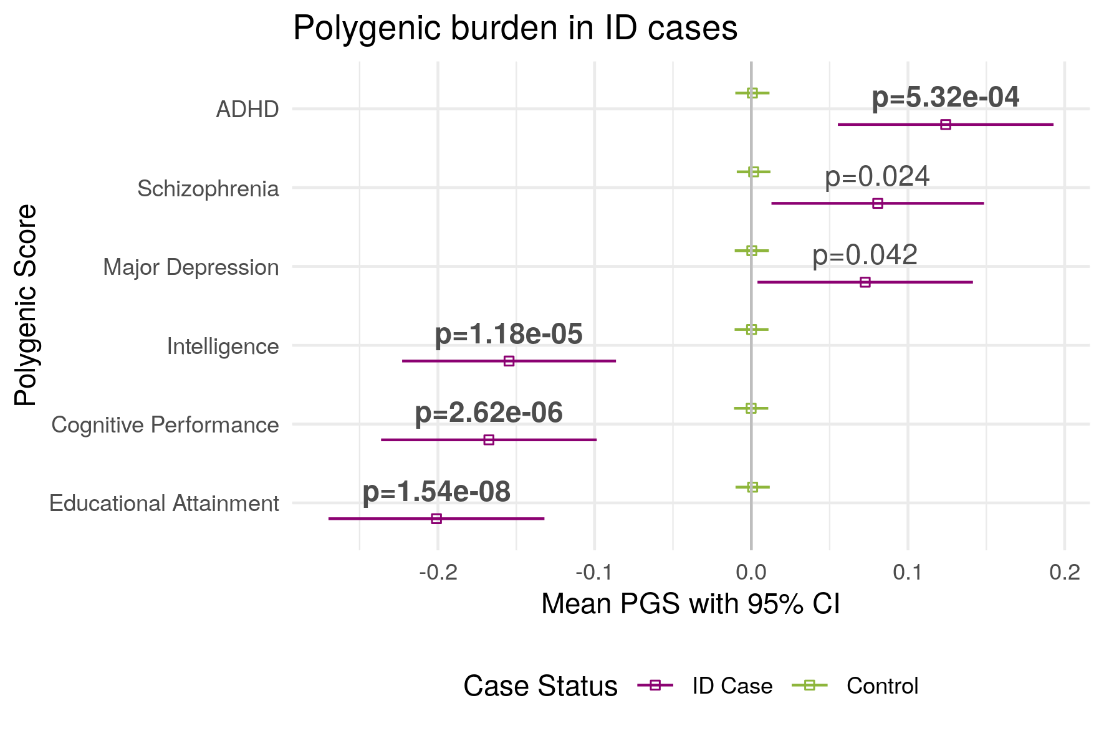


Supplementary Figure 7. Polygenic burden ID cases compared to population controls**.** Comparison of polygenic scores between intellectual disability cases and population controls. Scores are plotted as marginal mean polygenic scores with 95% confidence intervals. Bolded p values are those that pass Bonferroni multiple testing correction for 11 polygenic scores.


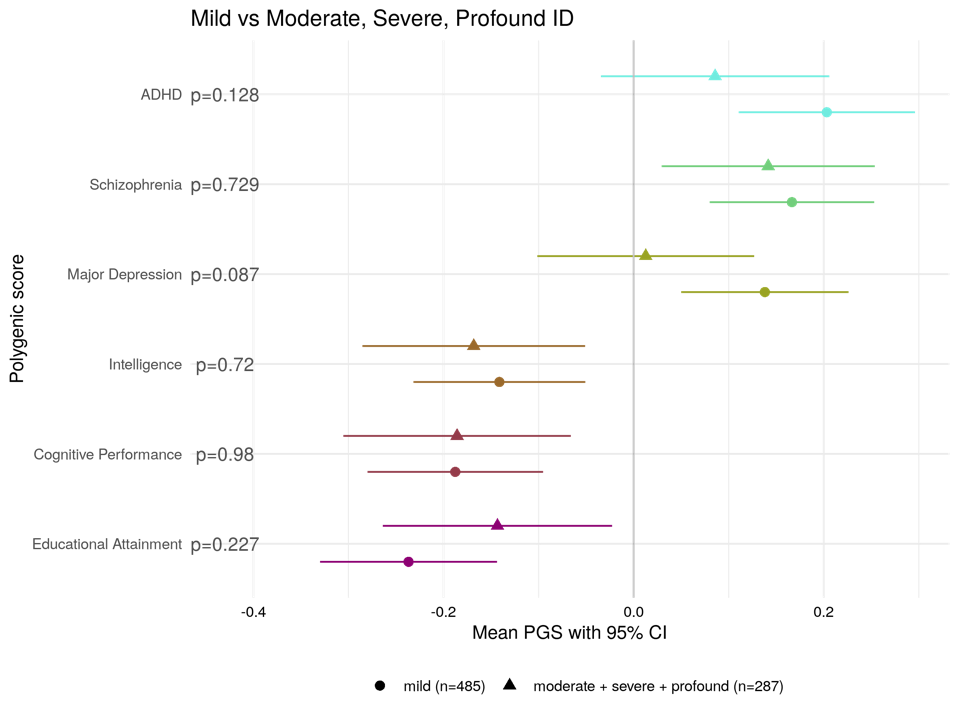


Supplementary Figure 8. Polygenic burden in mild vs more severe ID cases**.** Comparison of polygenic scores between mild intellectual disability cases and population controls. Scores are plotted as marginal mean polygenic scores with 95% confidence intervals. No comparisons passed Bonferroni multiple testing correction for 11 polygenic scores and 13 diagnostic phenotypes.


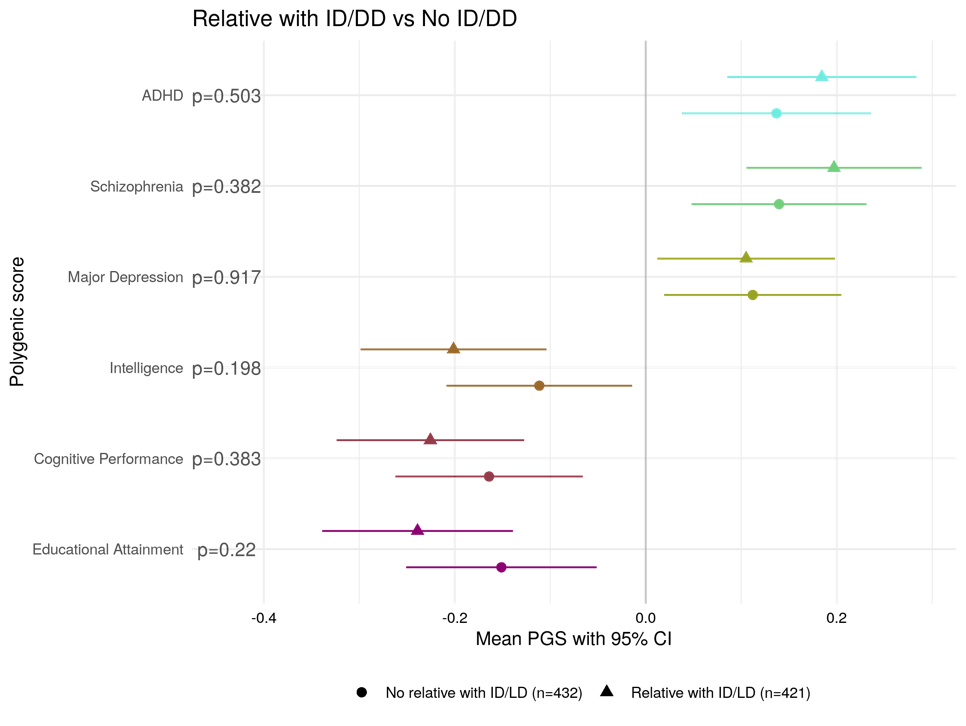


Supplementary Figure 9. Polygenic burden in individuals with a relative with ID or learning disability vs those without. Comparison of polygenic scores between ID cases with a reported relative with ID or a learning disability and those ID cases without a reported relative with ID or a learning disability. Scores are plotted as marginal mean polygenic scores with 95% confidence intervals. No comparisons passed Bonferroni multiple testing correction for 11 polygenic scores and 13 diagnostic phenotypes.


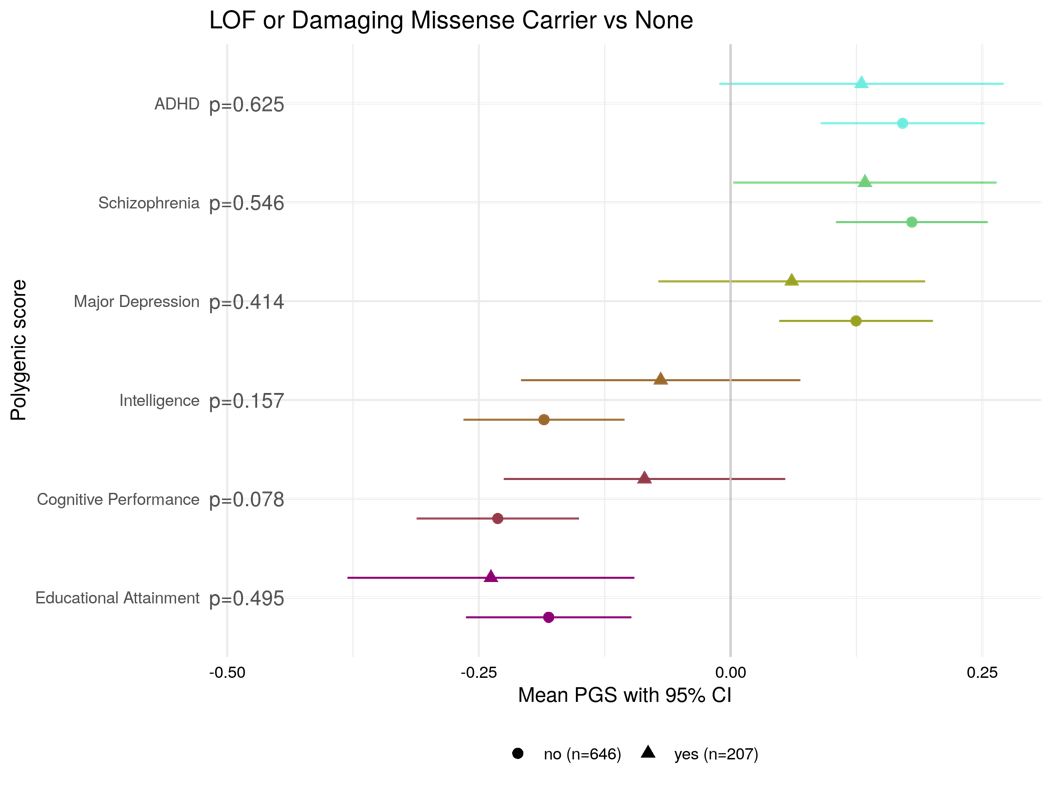


Supplementary Figure 10. Polygenic burden in rare variant carriers vs non-carriers**.** Comparison of polygenic scores between intellectual disability cases that are carriers of a rare, damaging variant (LOF or missense MPC > 2) in a known ID gene and those ID cases who are not carriers of a rare, damaging variant. Scores are plotted as marginal mean polygenic scores with 95% confidence intervals. No comparisons passed Bonferroni multiple testing correction for 11 polygenic scores and 13 diagnostic phenotypes.


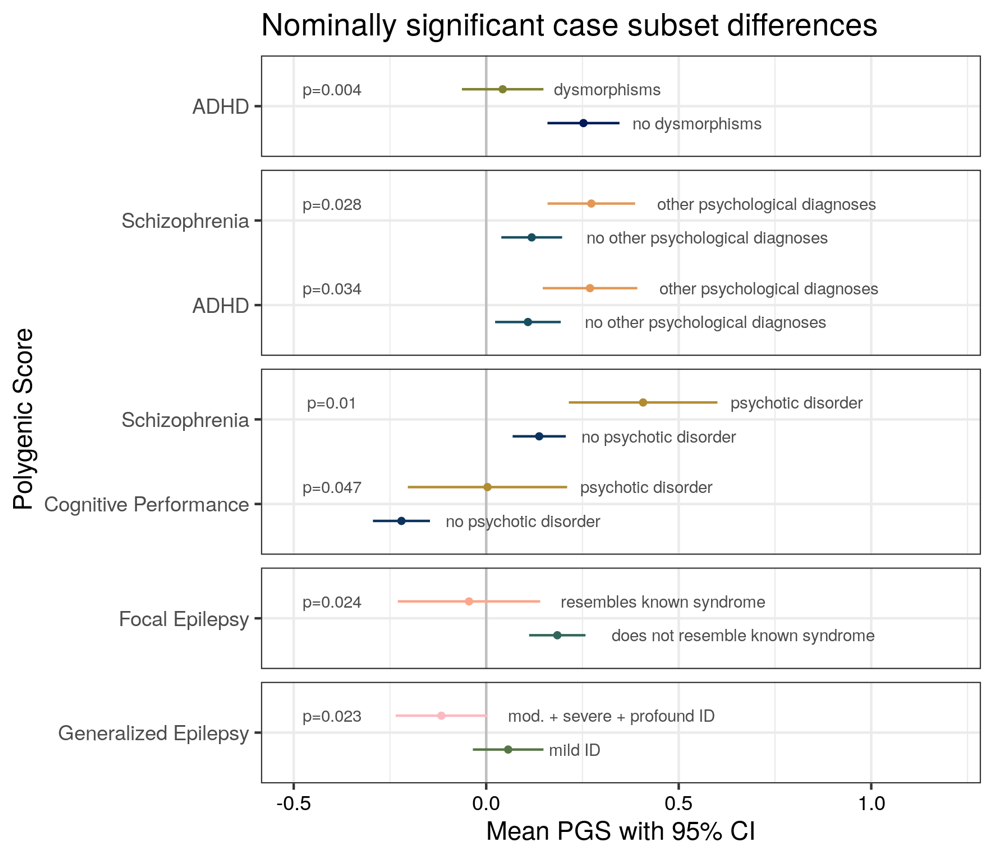


Supplementary Figure 11. Nominally significant differences in polygenic score between diagnostic ID case subsets. Comparison between polygenic scores between ID case diagnostic subsets, highlighting differences between case subset that were nominally significant on a particular polygenic score. Scores are plotted as marginal mean polygenic scores with 95% confidence intervals. No comparisons passed Bonferroni multiple testing correction for 11 polygenic scores and 12 diagnostic phenotype comparisons.


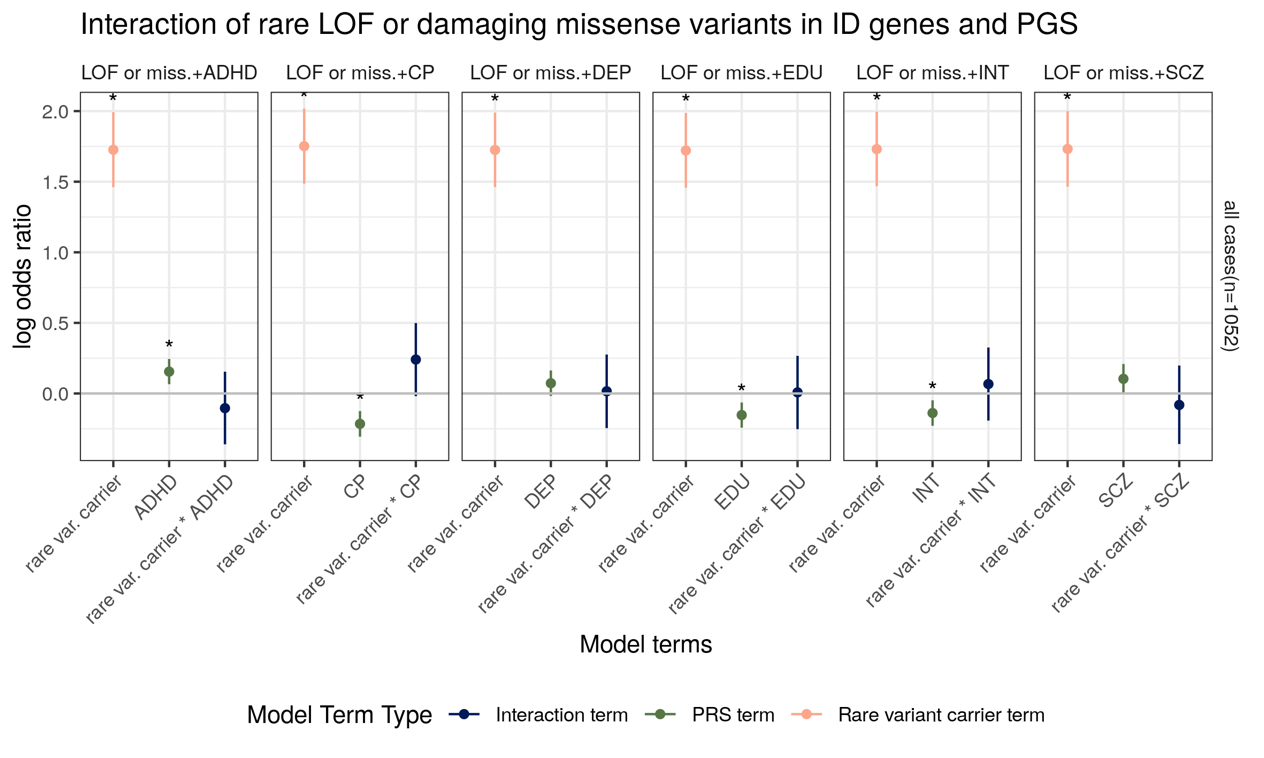


Supplementary Figure 12. Interaction between rare variants in known ID genes and polygenic score. Logistic regression comparing ID cases to population controls, including both LOF or missense MPC > 2 variant, polygenic score, and an interaction term between rare variant carrier status and polygenic score in the model. Each panel represents a different polygenic score included in the model with a fixed term for rare variant carrier status. ADHD = attention deficit hyperactivity disorder, CP = cognitive performance, DEP = major depression, EDU = education attainment, INT = intelligence, SCZ = schizophrenia. * indicates p < 0.05.


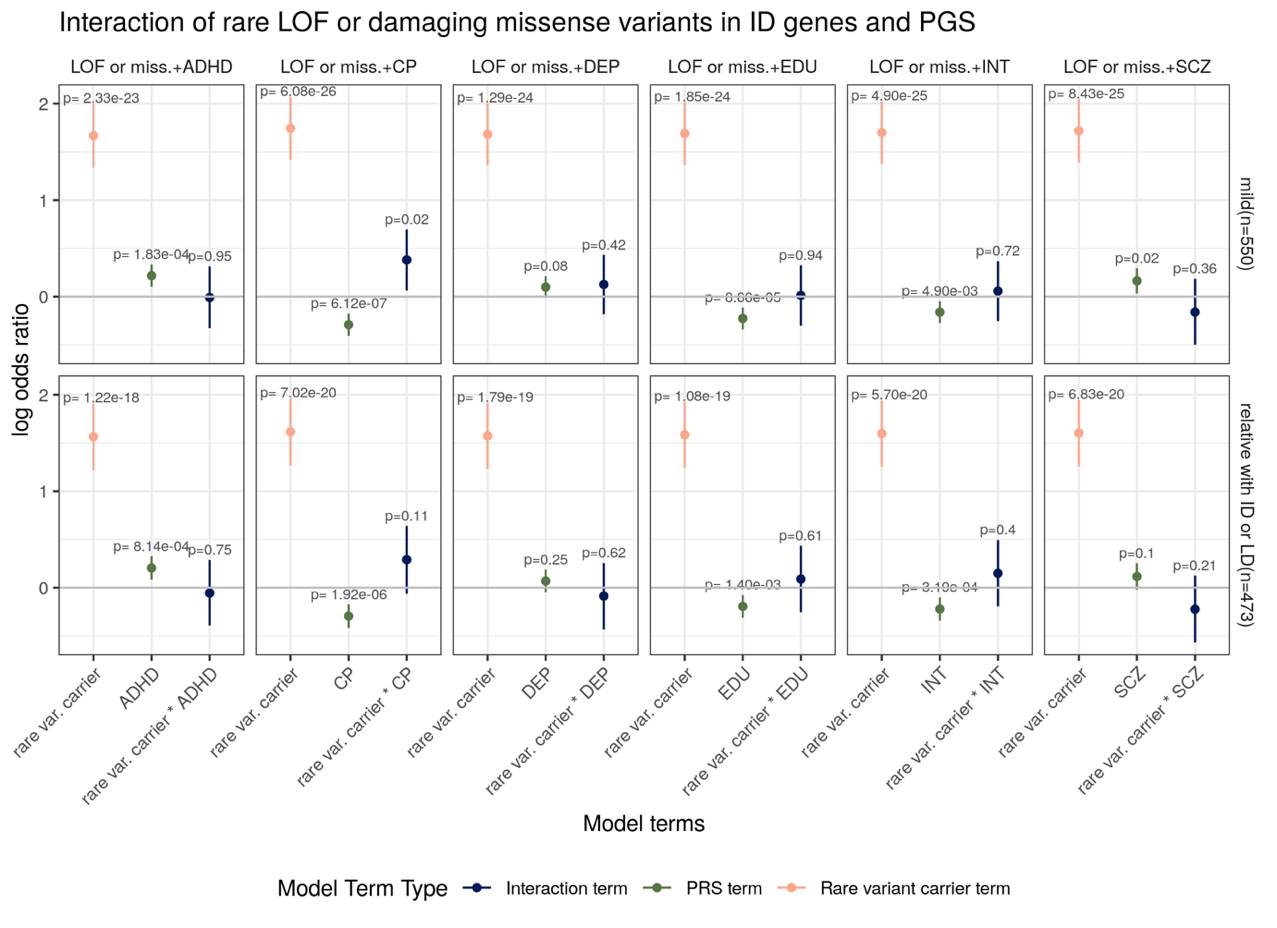


Supplementary Figure 13. Interaction between rare and common variants in cases with mild ID or a relative with ID**.** Logistic regression comparing ID case subsets to population controls, including both LOF and missense MPC > 2 variant carrier status, polygenic score, and an interaction term between rare variant carrier status and polygenic score in the model. Each panel represents a different polygenic score included in the model with a fixed term for damaging missense variant carrier status. ADHD = attention deficit hyperactivity disorder, CP = cognitive performance, DEP = major depression, EDU = education attainment, INT = intelligence, SCZ = schizophrenia.


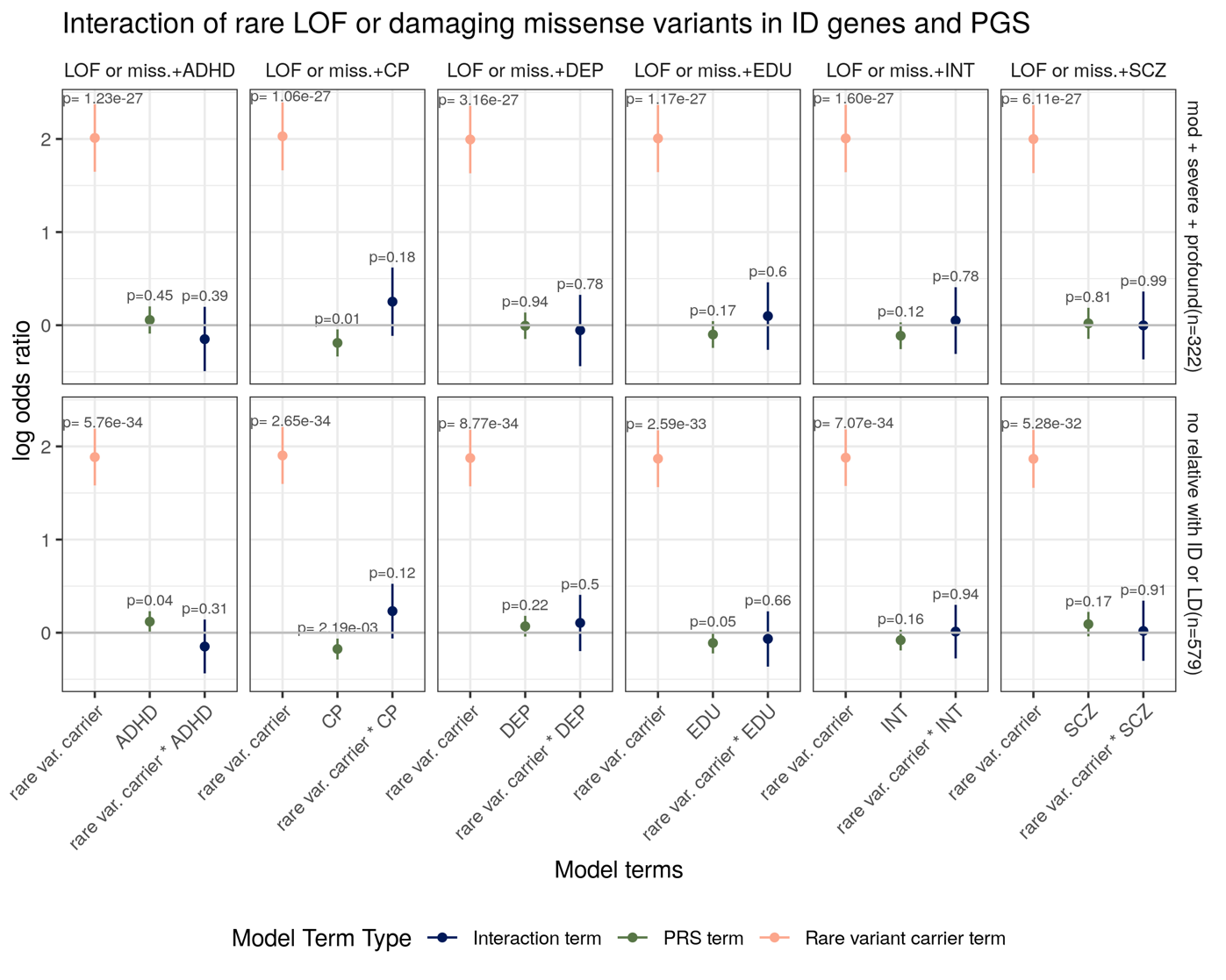


Supplementary Figure 14. Interaction between rare and common variants in cases with severe ID or without a relative with ID. Logistic regression comparing ID case subsets to population controls, including both LOF and missense MPC > 2 variant carrier status, polygenic score, and an interaction term between rare variant carrier status and polygenic score in the model. Each panel represents a different polygenic score included in the model with a fixed term for damaging missense variant carrier status. ADHD = attention deficit hyperactivity disorder, CP = cognitive performance, DEP = major depression, EDU = education attainment, INT = intelligence, SCZ = schizophrenia. **
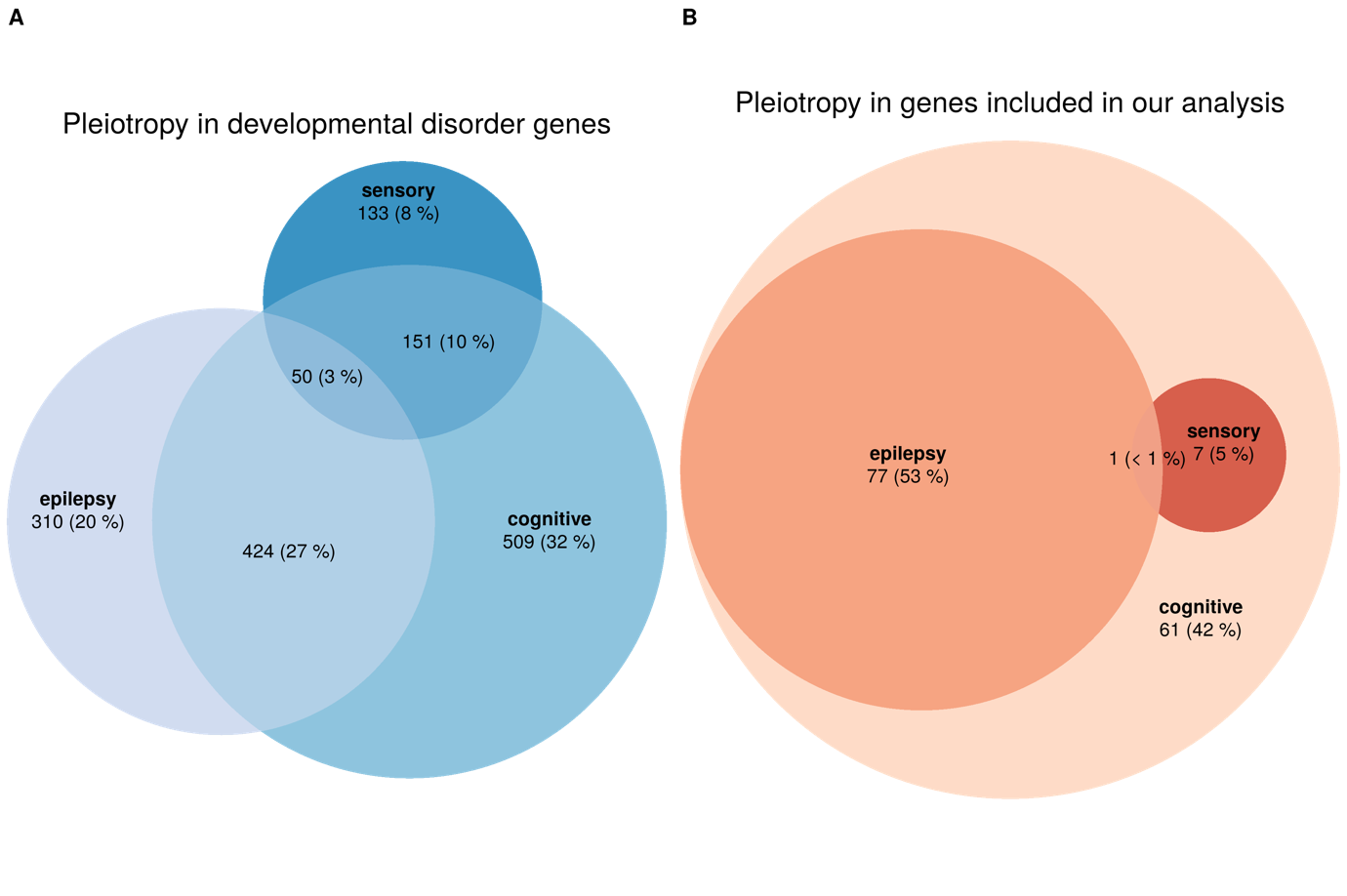
**

Supplementary Figure 15. Pleiotropy in high-confidence developmental disorder genes. A. Genes implicated in cognitive phenotypes (DECIPHER), sensory disabilities (DECIPHER), or early onset or syndromic epilepsy (Genomics England PanelApp). There is a high overlap of genes associated with all three disorders. Not pictured are 4 genes (<1%) implicated in epilepsy and sensory disorders, but not reduced cognition. B. Proportion of genes included in our rare variant burden analysis (Figure 1 and 2) that are also implicated in epilepsy or a sensory disorder.


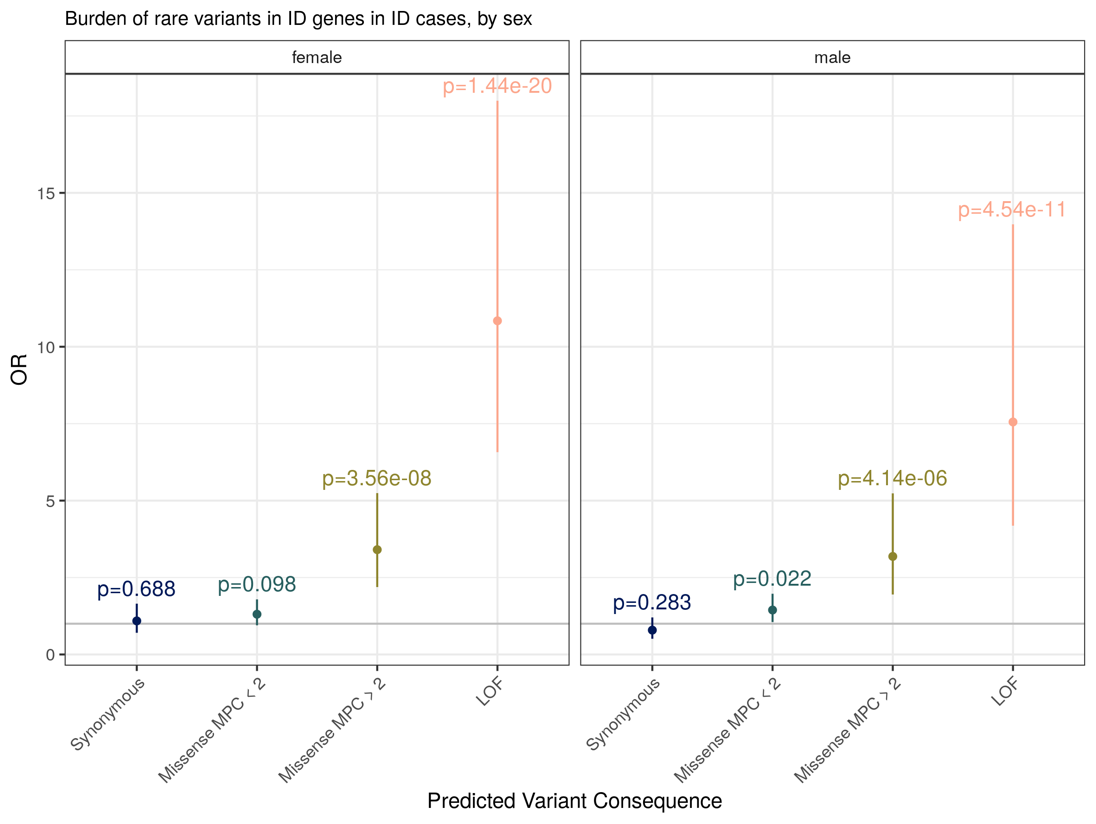


Supplementary Figure 16. Rare variant burden in known ID genes in males and females. Burden of rare, damaging heterozygous variants in known monoallelic ID genes in NFID cases (n female = 404, n male = 651) compared to population controls (n female = 2777, n male = 2014).


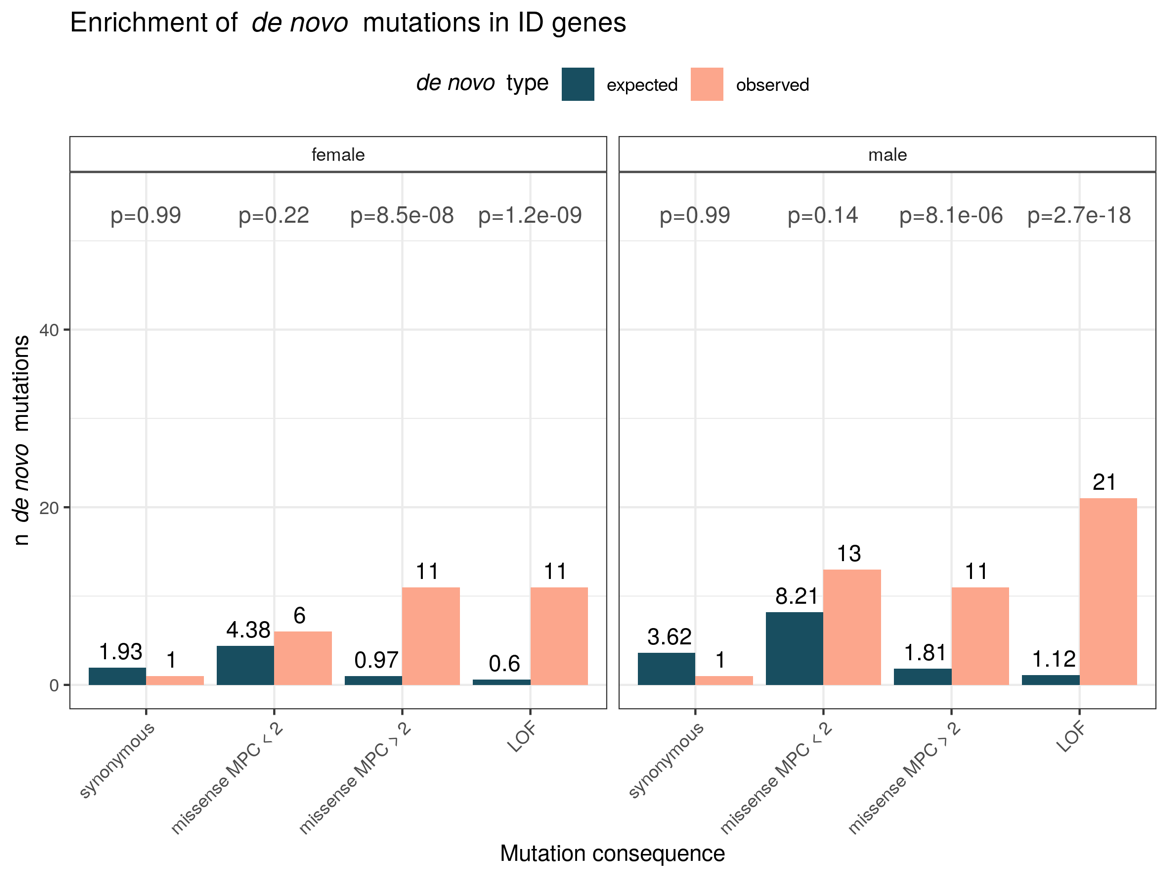


Supplementary Figure 17. Enrichment of *de novo* variants in known ID genes in males and females. Comparison of observed and expected *de novo* variants in known ID genes in ID cases (n female = 145, n male = 272).


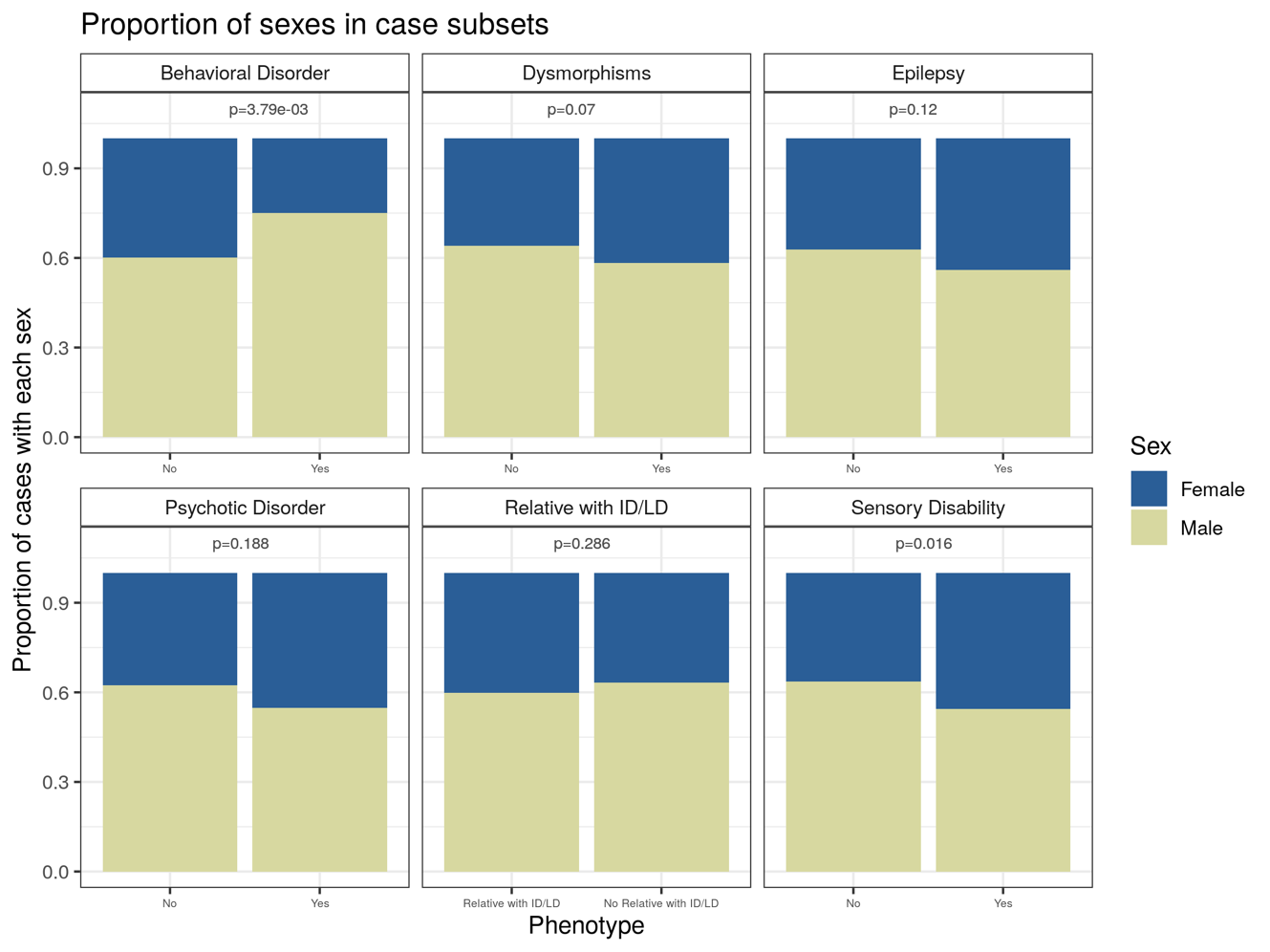


Supplementary Figure 18. Proportion of male and female cases in diagnostic subsets. Comparison of the proportion of male and female cases among case diagnostic subsets. P values represent Chi square test for difference of proportion of males and females between diagnostic case comparisons.

## Supplementary Tables

| Learning Disorders | |
| --- | --- |
| Speech comprehension or production disorder | n=209 |
| Mixed developmental disorder | n=65 |
| Learning disability | n=54 |
| Motor/movement disorder | n=11 |
| Behavioral Disorders | |
| Conduct/emotional disorder | n=51 |
| Unspecified behavioral disorder | n=39 |
| Attention disorder | n=25 |
| Tic disorder | n=16 |
| Psychological disorders | |
| Autism spectrum disorder | n=134 |
| Anxiety or depressive disorder | n=85 |
| Psychotic disorder | n=81 |
| Unspecified psychiatric disorder | n=18 |
| Mood disorder | n=18 |
| Compulsive disorder | n=11 |
| Post-traumatic/dissociative disorder | n=9 |
| Sleep disorder | n<5 |
| Substance abuse disorder | n<5 |
| Sensory disability | |
| Visual impairment | n=230 |
| Hearing impairment | n=29 |
| Other sensory impairment | n=6 |
| Dysmorphisms | |
| Abnormal facial features | n=201 |
| Teeth/palate/mouth abnormalities | n=72 |
| Skeletal abnormality | n=47 |
| Abnormal hand/foot development | n=44 |
| Macrocephaly | n=43 |
| Microcephaly | n=35 |
| Short stature | n=24 |
| Skin abnormalities | n=19 |
| Tall stature | n=8 |
| Obese or overweight | n=8 |
| Brain structure abnormality | n=7 |
| Underweight/low muscle mass | n=6 |
| Unspecified dysmorphism | n<5 |
| Internal organ dysmorphism | n<5 |

Supplementary Table 3. Patient phenotypes for binary categorical variables**.** Each phenotype was converted into a binary variable (“yes”, “no”) from free-text answers reported by physicians or care team members in the study questionnaires. Free-text answers were generalized to the descriptions for each patient above.

| Phenotype | Num. ID cases in each category | | Num. rare variants carriers | Heterozygous variant burden | Homozygous variant burden |
| --- | --- | --- | --- | --- | --- |
| ID severity | Mild ID | n=550 | n=84 | OR=1.95  (1.36-2.78 95% CI) | n.s. |
|  | Moderate, severe, profound ID | n=324 | n=82 |  |  |
| Relative with ID/LD | No relative with ID/LD | n=582 | n=124 | OR=1.85  (1.31-2.62 95% CI) | OR=0.34  (0.14-0.75 95% CI) |
|  | Relative with ID/LD | n=473 | n=63 |  |  |
| Psychotic Disorder | Psychotic disorder | n=93 | n=25 | OR=1.70  (1.01-2.77 95% CI) | n.s. |
|  | No psychotic disorder | n=962 | n=162 |  |  |
| Sensory Disability | Sensory disability | n=222 | n=61 | 0OR=2.13  (1.48-3.06 95% CI) | n.s. |
|  | No sensory disability | n=833 | n=126 |  |  |
| Dysmorphism | Dysmorphism | n=432 | n=98 | OR=1.73  (1.24-2.40 95% CI) | n.s. |
|  | No dysmorphism | n=623 | n=89 |  |  |
| Behavioral Disorder | Behavioral Disorder | n=108 | n=26 | OR=1.69  (1.01-2.75 95% CI) | n.s. |
|  | No behavioral disorder | n=947 | n=161 |  |  |

Supplementary Table 4. Burden of rare, damaging variants in known ID genes**.** Number of ID cases in each category and odds ratios with 95% confidence intervals for each phenotype.

| Phenotype | Num. ID cases in each category | | Num. rare variant carriers | *de novo* variant burden |
| --- | --- | --- | --- | --- |
| ID severity | Mild ID | n=236 | n = 24 | OR=2.20 (1.12-4.32 95% CI) |
|  | Moderate, severe, profound ID | n=103 | n = 22 |  |
| Relative with ID/LD | No relative with ID/LD | n=171 | n = 29 | OR=1.94 (1.08-4.39 95% CI) |
|  | Relative with ID/LD | n=279 | n = 28 |  |
| Epilepsy | Epilepsy | n=72 | n = 21 | OR=3.48 (1.75-6.84 95% CI) |
|  | No epilepsy | n=378 | n = 36 |  |
| Sensory Disability | Sensory disability | n=104 | n = 24 | OR=2.80 (1.51-5.16 95% CI) |
|  | No sensory disability | n=346 | n = 33 |  |

Supplementary Table 5. Enrichment of de novo variants in known ID genes**.** Number of ID cases (with both parents sequenced) in each category and odds ratios with 95% confidence intervals for each phenotype.

# Supplementary Methods

## Northern Finland Intellectual Disability cohort

Individuals were recruited from the Northern Ostrobothnia Hospital District’s Center for Intellectual Disability Care, the Department of Clinical Genetics of Oulu University Hospital, and pediatric neurology units and centers for intellectual disability care in the special responsibility area of Oulu University Hospital. Subjects with either intellectual disability (ICD-10 codes F70-79) or pervasive and specific developmental disorders (ICD-10 codes F80-89) of unknown etiology were included. Subjects with copy number variation of unknown clinical significance or highly variable phenotypes were also invited to participate in the study. Individuals of all ages were recruited for the study through routine visits to study centers or through identification through hospital records and invitation via mail. All subjects and/or their legal guardians provided written informed consent to participate in the study, and the ethical committees of the Northern Ostrobothnia Hospital District and the Hospital District of Helsinki and Uusimaa approved the study. DNA samples from the participants were extracted primarily from peripheral blood. In case peripheral blood could not be obtained, DNA was extracted from saliva.

Cases were clinically examined by multi-professional teams, which consisted of physicians, nurses, psychologists, physiotherapists, speech and occupational therapists, and social workers. Clinical diagnostic tests varied considerably depending on the subject’s clinical diagnosis, phenotype, and age. Standardized IQ tests included different versions of the following tests: Weschler Adult Intelligence Scale (WAIS), Weschler Intelligence Scale for Children (WISC), and Weschler Preschool and Primary Scale of Intelligence (WPPSI). Diagnosis of comorbid neuropsychiatric disorders were also based on multi-professional evaluation and clinical methods, such as ADOS (Autism Diagnostic Observation Schedule), ADI-R (Autism Diagnostic Interview), and CARS (Childhood Autism Rating Scale) for autism spectrum disorder and diagnosis of different forms of psychosis, including schizophrenia, were obtained from health care records and multi-professional evaluation.

## Population control individuals

Exome sequencing and DNA array genotype data for population control individuals were obtained from the FINRISK and Health2000-2011 studies, by permission from THL Biobank. FINRISK is a series of population-based health examination surveys carried out every five years since 1972 to monitor the risk of chronic diseases(1), with follow-up for disease endpoints using annual record linkage with the Finnish National Hospital Discharge Register and the National Causes-of-Death Register. The Health 2000 and 2011 studies are population-based health examination surveys carried out with the purpose of monitoring the health, functional capacity, and welfare of Finns as well as related service needs and use(2). Individuals in the FINRISK and Health2000-2011 studies that had known learning or psychiatric disorders as given in the study variables were excluded as controls.

## Exome sequencing and quality control

We first filtered variants and genotypes with relaxed thresholds to remove clearly poorly captured variants before samples filtering. We filtered all genotypes to those with a minimum depth (DP) of 10. For homozygous reference genotypes, we filtered to genotype calls with reference read ratio (reference allele depth/total allele depth) > 90% and a minimum Phred-scaled genotype quality of 20. For heterozygous genotypes, we filtered to genotype calls with reference read ratios between 20-80%. For homozygous alternate genotypes, we filtered to genotype calls with reference read ratio < 10%. For both heterozygous and homozygous alternate genotypes, we filtered to those with a minimum reference allele Phred-scaled genotype likelihood (PL ref) of 20, allowing retention of genotypes that have a high likelihood of not being a homozygous reference call but ambiguity between a heterozygous or homozygous alternate genotype call.

We removed variants not in the highest GATK VQSR tranche (0.98% of variants), with variant call rate less than 0.8 (41.07%), quality by depth less than 2 (for single-nucleotide polymorphisms, SNPs) or 3 (for insertions or deletions, indels) (13.44%), Hardy-Weinberg equilibrium test p value (calculated in controls only) less than 1e-9 (0.92%), mean depth < 10 (31.8%) or mean Phred-scaled genotype quality < 20 (30.25%). Additionally, we removed variants with more than 20% of heterozygous genotypes failing reference read ratio threshold (reference allele depth < 20% or > 80% of all reads, after filtering out genotypes failing depth and genotype quality measures) (10.37%). Reported percentages of variants failing on each measure reflect failing on that particular measure- many variants failed quality control on multiple measures.

We next filtered samples with chimera or contamination percentage more than 5% (n removed = 3), whose genetically-determined sex was ambiguous (F statistic between 0.4-0.8) (n=40), or who appeared to be related to more than 30 other samples in the dataset (indicating sample contamination) (n=167). Additionally, samples were separated into sequencing batches and individuals were removed who were greater or less than 4 standard deviations from the batch mean on: ratio of transition to transversion mutations (n=0), ratio of heterozygous to homozygous alternate mutations (n=52), ratio of insertions to deletions (n=0), or number of singleton (private) mutations (n=37). Population outlier samples were detected by iteratively calculating the first two principal components for all samples (after filtering to variants with minor allele frequency > 5% and LD pruning), removing individuals more than 4 standard deviations from the mean on either principal component and repeating until no samples fell outside the 4 standard deviation threshold on the first two principal components (n removed = 48). Kinship was calculated between all samples using King(3) (following filtering to minor allele frequency > 5% and LD pruning). Individuals were considered related if they had a kinship coefficient > 0.0883, the lower bound of a second-degree relative, and individuals were chosen to maximize the number of unrelated cases in the final dataset.

Finally, we filtered variants and genotypes with a strict set of thresholds to ensure high-quality variants for association analysis. We filtered genotypes with identical thresholds as above (genotypes with DP < 10, GQ or PL ref <20, reference read ratio <90% for homozygous reference, reference read ratio <20% or >80% for heterozygous, and reference read ratio >10% for homozygous alternate were filtered out). We then filtered out variants not in the highest GATK VQSR tranche (0.98%), quality by depth less than 2 (for SNPs) or 3 (for indels) (13.44%), Hardy-Weinberg test (in controls only) p-value less than 1e-6 (0.22%), call rate less than 0.9 (79.79%) and variants with more than 20% of heterozygous genotypes with reference read ratio <20% or >80% (10.37%). Additionally, to account for differences in exome captures kits between cases and controls, variant call rate was calculated separately in cases and controls and any variants with case-specific call rate < 0.95 (62.26%) or control-specific call rate < 0.95 (87.11%) were removed.

## Rarity filters for identifying likely pathogenic variants

For likely pathogenic dominant variants, we filtered to those with allele count less than 5 and no reported homozygous carriers in either our population control individuals or the Gnomad v2 variant database(4) in any population, in DECIPHER genes with ‘monoallelic autosomal’, ‘monoallelic X heterozygous’ or ‘monoallelic X hemizygous’ (in males only) allelic requirements. For likely pathogenic recessive variants, we filtered to those with allele frequency less than 1% in both Gnomad and population controls and less than 5 reported homozygous carriers in either Gnomad or our population controls, in DECIPHER genes with ‘biallelic autosomal’ allelic requirement. Recessive variants were considered Finnish-enriched if their allele frequency was at least 2-fold higher than the next highest population allele frequency in Gnomad, or absent entirely from Gnomad. Gnomad variants not passing Gnomad’s own quality control filters or that had a significant difference in allele frequency between Gnomad exomes and Gnomad genomes were not used in assessing variant rarity.

## DNA array data processing and quality control

NFID samples were genotyped in seven batches on the Illumina Infinium CoreExome or Global Screening Array DNA microarray chips. FINRISK population controls were genotyped in ten batches on the Illumina Infinium CoreExome, Global Screening Array, Human610-Quad, OmniExpress, or Affymetrix GeneChip chips. Health2000-2011 controls were genotyped in three batches on the Illumina Infinium CoreExome, Human610-Quad, or G4L chips.

To account for batch differences, we first performed quality control on each batch individually. We first removed ambiguous (A/T and C/G) SNPs and individuals with a mismatch between reported and genetically-determined sex. We filtered out variants with call rate < 0.95 and Hardy-Weinberg equilibrium p value < 1e-12, and samples with heterozygosity > 4 standard deviations from the batch mean and sample call rate < 0.95. Following within-batch quality control, we merged each batch to others with the same DNA microarray chip identity (e.g. all Global Screening Array chips). Illumina OmniExpress and G4L chips had sufficiently overlapping variant content to combine to a single batch. We then performed quality control on each combined set of data, filtering first the lowest quality variants with Hardy-Weinberg p value < 1e-12 and call rate < 0.95, before filtering samples with sample call rate < 0.95. Finally, we removed variants with Hardy-Weinberg p value < 1e-9 and variant call rate < 0.98, as well as variants with minor allele frequency < 1%. DNA array quality control was run in Plink 1.9(5).

Samples were then imputed in five separate batches, using the Sequencing Initiative Suomi (SISU)(6) v3 imputation panel containing 3,775 high coverage (25-30x) whole genome sequences of Finnish individuals. Imputation followed the FIMM sequencing informatics genotype imputation workflow v3.0 V2(7). Briefly, we input chip data and filtered to autosomal and X chromosomes, aligned variant alleles to the human reference genome to correct for dataset-specific reference/alternate variant flips, and compared variant frequencies between input data and the reference panel. We then removed variants where allele frequencies differed by more than 10% or the log2 fold change is >5 or <-5. Input data was then pre-phased with Eagle v2.3.5(8) and phased with Beagle v4.1(9). Following imputation, Impute2-like(10) INFO scores were calculated for each variant. After imputing data in separate batches for maximal sample and variant content per batch, we merged all imputed datasets and filtered out variants with INFO score below 0.7 in any imputation batch.

## Cognitive impairment pleiotropy

To assess per-gene pleiotropy between epilepsy, sensory disorders, and intellectual disability, we assessed publicly available data. We used DECIPHER developmental disorder genes (downloaded on March 18^th^, 2022, the same version used in the main analysis presented in this paper) and identified genes with strong or definitive evidence and reported organ specificities as “Brain/Cognition”. These cognitive impairment genes were the same ones used in the analysis reported in Figures 1 and 2. We additionally identified genes with reported “Eye” and “Ear” organ specificities, and more detailed phenotypes associated with these main organ malformations (e.g. “Eye: Lens”, “Eye: Retina”). We then joined this information with the Genomics England PanelApp list of early onset or syndromic epilepsy genes (version 4.144) to assess overlapping association with epilepsy (Supplementary Figure 15, Panel A).

To assess likely pleiotropy in our analysis, we compared DECIPHER genes and Genomics England PanelApp(11) early onset or syndromic epilepsy genes reported as associated with the phenotype in a monoallelic state and filtered to genes where a likely damaging variant was found in our cohort (Supplementary Figure 15, Panel B). Venn diagrams were created with the R package eulerr(12).

## References

1. Vartiainen E, Laatikainen T, Peltonen M, Juolevi A, Männistö S, Sundvall J, et al. Thirty-five-year trends in cardiovascular risk factors in Finland. Int J Epidemiol. 2010 Apr 1;39(2):504–18.

2. Gould R, Ilmarinen J, Järvisalo J, Koskinen S. Dimensions of work ability : Results of the Health 2000 Survey [Internet]. 2008 [cited 2022 Aug 25]. Available from: https://www.julkari.fi/handle/10024/78055

3. Robust relationship inference in genome-wide association studies | Bioinformatics | Oxford Academic [Internet]. [cited 2022 Aug 30]. Available from: https://academic.oup.com/bioinformatics/article/26/22/2867/228512

4. Karczewski KJ, Francioli LC, Tiao G, Cummings BB, Alföldi J, Wang Q, et al. The mutational constraint spectrum quantified from variation in 141,456 humans. Nature. 2020 May;581(7809):434–43.

5. Chang CC, Chow CC, Tellier LC, Vattikuti S, Purcell SM, Lee JJ. Second-generation PLINK: rising to the challenge of larger and richer datasets. GigaScience. 2015 Dec 1;4(1):s13742-015-0047–8.

6. Institute for Molecular Medicine Finland (FIMM), University of Helsinki, Finland. Sequencing Initiative Suomi project (SISu) [Internet]. Available from: http://sisuproject.fi

7. Pärn K, Isokallio MA, Fontarnau JN, Palotie A, Ripatti S, Palta P. protocols.io. 2019 [cited 2022 Sep 5]. Genotype imputation workflow v3.0. Available from: https://www.protocols.io/view/genotype-imputation-workflow-v3-0-xbgfijw

8. Loh PR, Danecek P, Palamara PF, Fuchsberger C, A Reshef Y, K Finucane H, et al. Reference-based phasing using the Haplotype Reference Consortium panel. Nat Genet. 2016 Nov;48(11):1443–8.

9. Browning BL, Browning SR. Genotype Imputation with Millions of Reference Samples. Am J Hum Genet. 2016 Jan 7;98(1):116–26.

10. Howie B, Marchini J, Stephens M. Genotype Imputation with Thousands of Genomes. G3 GenesGenomesGenetics. 2011 Nov 1;1(6):457–70.

11. Martin AR, Williams E, Foulger RE, Leigh S, Daugherty LC, Niblock O, et al. PanelApp crowdsources expert knowledge to establish consensus diagnostic gene panels. Nat Genet. 2019 Nov;51(11):1560–5.

12. Larsson J. eulerr: Area-Proportional Euler and Venn Diagrams with Ellipses [Internet]. 2022. Available from: https://CRAN.R-project.org/package=eulerr
